# (Nano)platforms in bladder cancer therapy: Challenges and opportunities

**DOI:** 10.1002/btm2.10353

**Published:** 2022-06-17

**Authors:** Milad Ashrafizadeh, Ali Zarrabi, Hassan Karimi‐Maleh, Afshin Taheriazam, Sepideh Mirzaei, Mehrdad Hashemi, Kiavash Hushmandi, Pooyan Makvandi, Ehsan Nazarzadeh Zare, Esmaeel Sharifi, Arul Goel, Lingzhi Wang, Jun Ren, Yavuz Nuri Ertas, Alan Prem Kumar, Yuzhuo Wang, Navid Rabiee, Gautam Sethi, Zhaowu Ma

**Affiliations:** ^1^ Faculty of Engineering and Natural Sciences Sabanci University, Orta Mahalle Istanbul Turkey; ^2^ Department of Biomedical Engineering, Faculty of Engineering and Natural Sciences Istinye University Istanbul Turkey; ^3^ School of Resources and Environment University of Electronic Science and Technology of China Chengdu People's Republic of China; ^4^ Department of Chemical Engineering Quchan University of Technology Quchan Iran; ^5^ Department of Chemical Sciences University of Johannesburg Johannesburg South Africa; ^6^ Department of Orthopedics, Faculty of medicine Tehran Medical Sciences, Islamic Azad University Tehran Iran; ^7^ Farhikhtegan Medical Convergence Sciences Research Center Farhikhtegan Hospital Tehran Medical Sciences, Islamic Azad University Tehran Iran; ^8^ Department of Biology, Faculty of Science Islamic Azad University, Science and Research Branch Tehran Iran; ^9^ Department of Food Hygiene and Quality Control, Division of epidemiology, Faculty of Veterinary Medicine University of Tehran Tehran Iran; ^10^ Istituto Italiano di Tecnologia Centre for Materials Interface Pontedera Pisa 56025 Italy; ^11^ School of Chemistry Damghan University Damghan Iran; ^12^ Department of Tissue Engineering and Biomaterials, School of Advanced Medical Sciences and Technologies Hamadan University of Medical Sciences Hamadan Iran; ^13^ La Canada High School La Cañada Flintridge California USA; ^14^ Cancer Science Institute of Singapore National University of Singapore Singapore Singapore; ^15^ Department of Laboratory Medicine and Pathology University of Washington Seattle Washington USA; ^16^ Shanghai Institute of Cardiovascular Diseases, Department of Cardiology Zhongshan Hospital, Fudan University Shanghai China; ^17^ Department of Biomedical Engineering Erciyes University Kayseri Turkey; ^18^ ERNAM—Nanotechnology Research and Application Center Erciyes University Kayseri Turkey; ^19^ Department of Pharmacology Yong Loo Lin School of Medicine, National University of Singapore Singapore Singapore; ^20^ Department of Urologic Sciences and Vancouver Prostate Centre University of British Columbia Vancouver British Columbia Canada; ^21^ School of Engineering Macquarie University Sydney New South Wales 2109 Australia; ^22^ Department of Materials Science and Engineering Pohang University of Science and Technology (POSTECH) Pohang Gyeongbuk 37673 South Korea; ^23^ Health Science Center Yangtze University Jingzhou Hubei China

**Keywords:** bladder cancer, clinical application, drug delivery, phototherapy, smart nanocarriers

## Abstract

Urological cancers are among the most common malignancies around the world. In particular, bladder cancer severely threatens human health due to its aggressive and heterogeneous nature. Various therapeutic modalities have been considered for the treatment of bladder cancer although its prognosis remains unfavorable. It is perceived that treatment of bladder cancer depends on an interdisciplinary approach combining biology and engineering. The nanotechnological approaches have been introduced in the treatment of various cancers, especially bladder cancer. The current review aims to emphasize and highlight possible applications of nanomedicine in eradication of bladder tumor. Nanoparticles can improve efficacy of drugs in bladder cancer therapy through elevating their bioavailability. The potential of genetic tools such as siRNA and miRNA in gene expression regulation can be boosted using nanostructures by facilitating their internalization and accumulation at tumor sites and cells. Nanoparticles can provide photodynamic and photothermal therapy for ROS overgeneration and hyperthermia, respectively, in the suppression of bladder cancer. Furthermore, remodeling of tumor microenvironment and infiltration of immune cells for the purpose of immunotherapy are achieved through cargo‐loaded nanocarriers. Nanocarriers are mainly internalized in bladder tumor cells by endocytosis, and proper design of smart nanoparticles such as pH‐, redox‐, and light‐responsive nanocarriers is of importance for targeted tumor therapy. Bladder cancer biomarkers can be detected using nanoparticles for timely diagnosis of patients. Based on their accumulation at the tumor site, they can be employed for tumor imaging. The clinical translation and challenges are also covered in current review.

## Graphical abstract

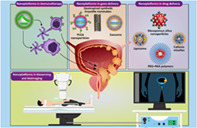

**Abbreviations**
2DMTT‐BBTD: D–*π*–A–*π*–D typed small molecule
AE: glycoalkaloidic extract
BBB: blood–brain barrier
BCG: Bacillus Calmette‐Guerin
BSA: bovine serum albumin
BTB: blood‐tumor barrier
CaP: calcium phosphate
CAR: chimeric antigen receptor
CC: calcium carbonate
CDT: chemodynamic therapy
Ce6: chlorin e6
circRNAs: circular RNAs
CM: chitosan‐polymethacrylic acid
CP: cisplatin
CRISPR: clustered regularly interspaced short palindromic repeats
CT: computed tomography
CTLA‐4: cytotoxic T‐lymphocyte‐associated protein 4
DOX: doxorubicin
DPP: 1,2‐dioleoyl‐3‐trimethylammonium propane/methoxypoly(ethyleneglycol)
EMVs: exosome‐mimetic nanovesicles
EVs: extracellular vesicles
FePPy‐NH_2_
: Fe(III)‐doped polyaminopyrrole
GA: gambogic acid
GCDs: guanidine‐terminated carbosilane dendrimers
GOx: glucose oxidase
GSH: glutathione
GZIF: glucose oxidase (GOx)‐encapsulated ZIF‐8
HA: hyaluronic acid
HAD: hyaluronic acid dialdehyde
HCPT: 10‐Hydroxycamptotechin
HRP: horseradish peroxidase
HSP90: heat shock protein 90
IAA: indole‐3‐acetic acid
MAA: methacrylic acid
MIBC: muscle‐invasive bladder cancer
MIP: molecularly imprinted polymers
miRs: microRNAs
MMPs: matrix metalloproteinases
MOFs: metal–organic frameworks
MRI: magnetic resonance imaging
MSCs: mesenchymal stem cells
MSNs: mesoporous silica nanoparticles
ncRNAs: noncoding RNAs
NDs: nanodiamonds
NIR: near‐infrared
NMIBC: nonmuscle invasive bladder cancer
NMP22: nuclear matrix protein 22
Nrf2: nuclear factor‐erythroid 2‐related factor 2
NTZ: nitazoxanide
NuMA1: nuclear mitotic apparatus protein 1
PAA: poly(l‐aspartic acid sodium salt)
PD1: programmed death‐1
PD‐L1: programmed death‐ligand 1
PDT: photodynamic therapy
PEG–PAA: methoxy‐poly(ethylene glycol)‐block‐PAA
PEI: polyethyleneimine
P‐gp: P‐glycoprotein
PTT: phototherapy
QDs: quantum dots
RAFT: reversible addition‐fragmentation chain transfer
RNAi: RNA interference
ROS: reactive oxygen species
SCNTs: synthetic chrysotile nanotubes
shRNA: short hairpin RNA
siRNA: small interfering RNA
SPIONs: superparamagnetic nanoparticles
SWCNTs: single‐walled carbon nanotubes
TITC: twisted intermolecular charge transfer
TME: tumor microenvironment
ZnO: zinc oxide

## 1 INTRODUCTION

Urological cancers are responsible for high mortality around the world, and bladder cancer is the most common tumor in the urinary system. There are two major forms of bladder cancer including muscle‐invasive bladder cancer (MIBC) and nonmuscle invasive bladder cancer (NMIBC). Incidence of metastasis in NMIBC is rare, albeit the chance of recurrence is 50%–70% after transurethral resection.^1^ Approximately 10%–20% of cases are MIBC, and risk of metastasis is high in certain MIBC patients which is responsible for short survival in nearly up to 30% of patients.^2^, ^3^ Following a radical cystectomy, some MIBC patients demonstrate lymphatic metastasis that can lead to mortality within 5 years after diagnosis.^4^ Most of the bladder cancer cases occur in men. In the US, the number of these patients is estimated to be 83,730.^5^, ^6^ In the middle‐aged and older adult men, bladder cancer is the second most common malignancy.^7^ There are several risk factors for initiation and development of bladder cancer including smoking, age, family history, exposure to chemicals and dyes, and chronic bladder inflammation.^8^ The prognosis of patients with NMIBC is desirable and by local treatment, the transformation of NMIBC to MIBC is prevented and on time therapy inhibits tumor recurrence. Due to the development of metastasis, the prognosis of patients with MIBC is unfavorable and in addition to resection, patients receive adjuvant chemotherapy.^9^ However, treatment appears not to be completely effective and patients present development of metastasis in spite of aggressive therapy.^10^ In addition to surgery and chemotherapy, radiotherapy and immunotherapy in certain cases can be employed for the treatment of bladder cancer.^11^, ^12^ There is also a classification of bladder cancer based on the manner of tumor development including squamous cell carcinoma and carcinoma in mucus‐secreting cells of the bladder which indeed is rare.^13^ Despite various therapeutic strategies for bladder cancer, tumor cells have capacity of developing resistance to therapy.^14^, ^15^ Therefore, novel modalities for treatment of bladder cancer should be followed. Furthermore, different diagnostic tools including magnetic resonance imaging (MRI), computed tomography (CT) and chest X‐ray, among others are utilized for bladder cancer detection. However, they also have their drawbacks such as low specificity and heterogeneous nature of bladder tumor cells.^16^

Radical cystectomy is the gold standard for bladder cancer treatment. Despite the malignancy of tumor cells, the 5‐year survival rate of patients with bladder cancer is estimated to be 20%–40%.^17^, ^18^ In order to improve the potential of therapy for bladder cancer, combination therapies with methotrexate, vinblastine, doxorubicin, and cisplatin are recommended.^19^ However, in high‐risk patients that have undergone radical cystectomy, the combination of cisplatin and gemcitabine for treatment of bladder cancer and improving overall survival is not significant.^19^ Based on estimates, it has been reported that only 50% of bladder cancer patients respond to cisplatin‐mediated chemotherapy.^20^ Compared to other regimens, the combination of cisplatin and gemcitabine demonstrates low side effects and a high potential in the treatment of bladder cancer and retardation of metastasis.^21^, ^22^ In addition to chemotherapy and radical cystectomy, pelvic lymph node dissection is also performed for treatment of bladder cancer.^23^, ^24^ For patients that undergo surgery, radiotherapy is also used as a treatment modality, and in combination with chemotherapy, effectiveness of the therapy increases.^25^ Despite significant efforts in the application of radiotherapy and chemotherapy as well as surgery in bladder cancer treatment, there are still challenges due to adverse impacts, resistance development, and metastatic nature of tumor cells. Metastasis of bladder tumor cells limits the application of surgery in bladder cancer treatment. Both radiotherapy and chemotherapy exhibit pronounced side effects in major organs. The potential of radiotherapy and chemotherapy in bladder cancer suppression is restricted by resistance development.^21^, ^26^, ^27^ One of the new emerging therapeutic approaches in bladder cancer is the application of immunotherapy. In recent years, various kinds of checkpoint inhibitors including PD‐L1/PD1, CTLA‐4 as well as CAR T cell therapy have been developed for bladder cancer. However, immune evasion has reduced potential of immunotherapy in bladder cancer.^28^ Nanotechnology is an interdisciplinary field that has opened the way for treatment and diagnosis of various diseases such as infectious diseases and cancer.^29^, ^30^, ^31^, ^32^, ^33^, ^34^, ^35^, ^36^, ^37^, ^38^, ^39^ Nanostructures possess sizes at nm ranges and therefore, they can be easily internalized by tumor cells. Nanostructures can be employed for the purpose of cargo (drug and gene) delivery, immunotherapy, phototherapy, and diagnosis. The purpose of current review is to provide a comprehensive discussion of applications of nanomaterials in bladder cancer therapy and diagnosis.

## 2 NANOPLATFORMS IN CHEMOTHERAPEUTIC DRUG DELIVERY

One of the optimal treatment approaches in cancer therapy is the application of chemotherapeutic agents. Intravenous injection is the main approach for the administration of these anti‐cancer drugs. However, systemic toxicity is mainly followed upon the application of chemotherapeutic agents, and it is recommended to use a low amount of these agents. Regardless of the adverse impacts of chemotherapeutic agents, frequent applications of these agents lead to the emergence of drug resistance. Therefore, solutions to these issues should be provided. In the first step, low concentration of chemotherapeutic agents should be employed and in the second step, combination therapy and increased accumulation of drugs at tumor site should be followed to prevent drug resistance. Emerging nanostructures have provided a great opportunity for delivery of chemotherapeutic agents to reduce their concentration (low level is loaded on nanocarriers), provide their targeted delivery, enhance their internalization in cancer cells, and prevent development of drug resistance.^40^, ^41^, ^42^ This section focuses on nano‐scale delivery systems for delivery of chemotherapeutic agents in bladder cancer therapy.

Among various kinds of chemotherapeutic agents employed for bladder cancer treatment, doxorubicin (DOX) is the most well‐known medication capable of suppressing tumor progression via binding to topoisomerase enzymes to counter DNA replication and cancer cell proliferation.^43^, ^44^, ^45^ Mesoporous silica nanoparticles (MSNs), modified with polydopamine and peptide, have been used for delivery of DOX. Polydopamine modification of MSNs is of importance for providing sustained release of DOX and its pH‐sensitive release, while peptide provides specific targeting of bladder tumor cells by binding to receptor. The drug‐loaded MSNs had a particle size of 170.2 nm, zeta potential of −15.9 mV, and drug loading of 16.25%. These nanoparticles demonstrated high internalization capacity in bladder tumor cells, and they improved potency of DOX in bladder tumor suppression in vivo.^46^ An interesting point is the increased anti‐cancer activity of DOX against bladder tumors and reduced adverse impacts following encapsulation of DOX using nanostructures.^47^

Lipid‐based nanostructures are also promising candidates for DOX delivery in bladder cancer. Cationic micelles were prepared from 1,2‐dioleoyl‐3‐trimethylammonium propane/methoxypoly (ethyleneglycol) (DPP) for DOX delivery and resulting nanocarriers were 18.65 nm in size and +19.6 mV in zeta potential. The cellular uptake and accumulation of DOX in bladder tumor cells were significantly enhanced, and these nanoparticles promoted residence of DOX in bladder. The in vivo experiment revealed boosted anti‐cancer activity of DOX against bladder tumor.^48^ In addition to micelles, other kinds of lipid‐based nanostructures, known as liposomes, can be employed in DOX delivery. At the first step, folate‐modified thermosensitive liposomes were fabricated and then, DOX, gold nanorods, and magnetic nanoparticles were loaded into these liposomes. This nanocomplex showed a particle size of 230 nm with superparamagnetic features capable of loading 0.57 mg/ml of DOX. The photothermal impact and alterations in temperature affect the release of DOX from these liposomal nanoparticles. After irradiation, 95% of DOX was released in 3 h, and due to modification with folic acid, the nanostructures bound to folate receptors on the surface of bladder cancer cells, resulting in an increase in their internalization.^49^

Nanodiamonds (NDs) are a kind of solid nanostructures deemed ideal candidates for drug delivery and biomedical application courtesy of their high surface area and high drug loading efficiency.^50^, ^51^, ^52^ The availability of NDs has opened a new window in cancer treatment due to their capacity in reversing chemoresistance.^52^, ^53^ However, the stability of NDs is a troublesome problem limiting their potential in cargo delivery.^54^, ^55^ It has been reported that coating NDs with polymers is a promising strategy to improve their stability. Chitosan‐modified NDs were synthesized for intravesical delivery of DOX in bladder cancer therapy. These nanostructures demonstrated high drug loading efficiency (more than 90%) with an average particle size of 150 nm. The chitosan‐modified NDs present great colloidal stability and desirable drug release, but their stability is relatively low in cultured media. These DOX‐loaded chitosan‐modified NDs significantly suppressed bladder tumor progression ex vivo and provided high drug retention.^56^ In fact, the ultimate goal of nanoscale delivery for DOX is to improve and to prolong its retention in bladder tumor site to decrease tumor cell viability up to 99%.^57^

Another option in bladder cancer chemotherapy is cisplatin (CP) which is commonly used for locally advanced or metastatic tumors.^22^ However, clinical trials show limitations of using CP for purpose of bladder cancer chemotherapy and to overcome the drawbacks, intravesical delivery of CP is suggested. CP‐loaded nanostructures have shown potential for treatment of bladder cancer in clinical trials and reducing the adverse impacts of CP.^58^, ^59^, ^60^, ^61^, ^62^, ^63^, ^64^, ^65^ Coating nanomaterials with PEG enhances local delivery of CP and improves survival of animal models.^66^ In an effort, CP was conjugated to poly(l‐aspartic acid sodium salt) (PAA) or methoxy‐poly(ethyleneglycol)‐block‐PAA (PEG–PAA) polymers with low or high PEG content. The intravesical administration of these nanoparticles significantly diminished side effects of CP and improved its safety profile. It is worth mentioning that administration of CP is correlated with hyperplasia and overweight of bladder tissue, while CP‐loaded nanocarriers demonstrated high biocompatibility and lack of such drawbacks. CP‐loaded nanocarriers had a particle size of 140 nm, zeta potential of −3.3 mV with drug loading up to 40%. These nanoparticles demonstrated great anti‐proliferative activity against bladder cancer. Notably, CP‐loaded PAA‐modified nanostructures increased CP concentration in bladder tissue, while PEG‐modified nanoparticles had no effect.^67^

Polymeric nanoparticles and their combination with superparamagnetic iron oxide nanostructures (SPIONs) can be employed for CP delivery in bladder cancer therapy to offer an adjusted release. First, PCL‐b‐P(PMA‐click‐MSA‐co‐PEGMA) nanoparticles were synthesized using three main strategies including ring‐opening polymerization, reversible addition‐fragmentation chain transfer (RAFT) polymerization, and thiol‐yne “click” reaction. At the next step, SPIONs were embedded into PCL core, whereas CP was conjugated to surface of nanostructures via binding to dicarboxylic groups. These nanoparticles with a particle size and zeta potential of 281 nm and −34.3 mV, respectively, demonstrated mucoadhesive and superparamagnetic characteristics. After the administration of CP‐loaded polymeric nanoparticles, 30% drug release (CP) occurred during first 4 h and then, prolonged release of CP was maintained for 4 days. The increase in temperature promoted the release of CP from nanostructures and they showed high anti‐cancer activity against bladder tumor.^68^ In addition to drug delivery, several nanoparticles such as cuprous oxide nanostructures have anti‐tumor activity and increase ROS generation. These nanoparticles evoke apoptosis in reducing progression of bladder tumor cells, providing a synergy with chemotherapeutic agents such as CP and gemcitabine.^69^ Therefore, targeted delivery of anti‐cancer agents by nanostructures significantly promotes their anti‐proliferative activity in bladder cancer.^70^, ^71^, ^72^

Interestingly, nanocarriers can provide a platform for co‐delivery of chemotherapeutic agents. Several experiments have revealed that co‐delivery of synthetic drugs with nanostructures provides synergistic therapy in bladder cancer. Chitosan‐polymethacrylic acid (CM) nanocapsules with capacity of attaching to luminal surface of bladder tissue and lack of damage to urothelium showed high biocompatibility and safety profile. These nanocapsules were prepared via electrostatic interaction between chitosan and methacrylic acid (MAA) chains. Then, CP and DOX as anti‐cancer agents were loaded on these nanoparticles. This combination demonstrated 5‐ to 16‐fold increase in anti‐cancer activity compared to CP or DOX alone, and they could be internalized in bladder tumor cells with a high efficiency. DOX‐ and CP‐loaded nanocapsules had a zeta potential of +15 mV, confirming their high stability.^73^ Usually, nanoparticles employed for co‐delivery of synthetic drugs have a high encapsulating efficiency (70% or more), and the only limitation is the increase in particle size due to co‐loading, necessitating the need for the understanding of how enhancement in particle size can affect internalization of nanocarriers in bladder cancer cells.^74^, ^75^

**FIGURE 1:**
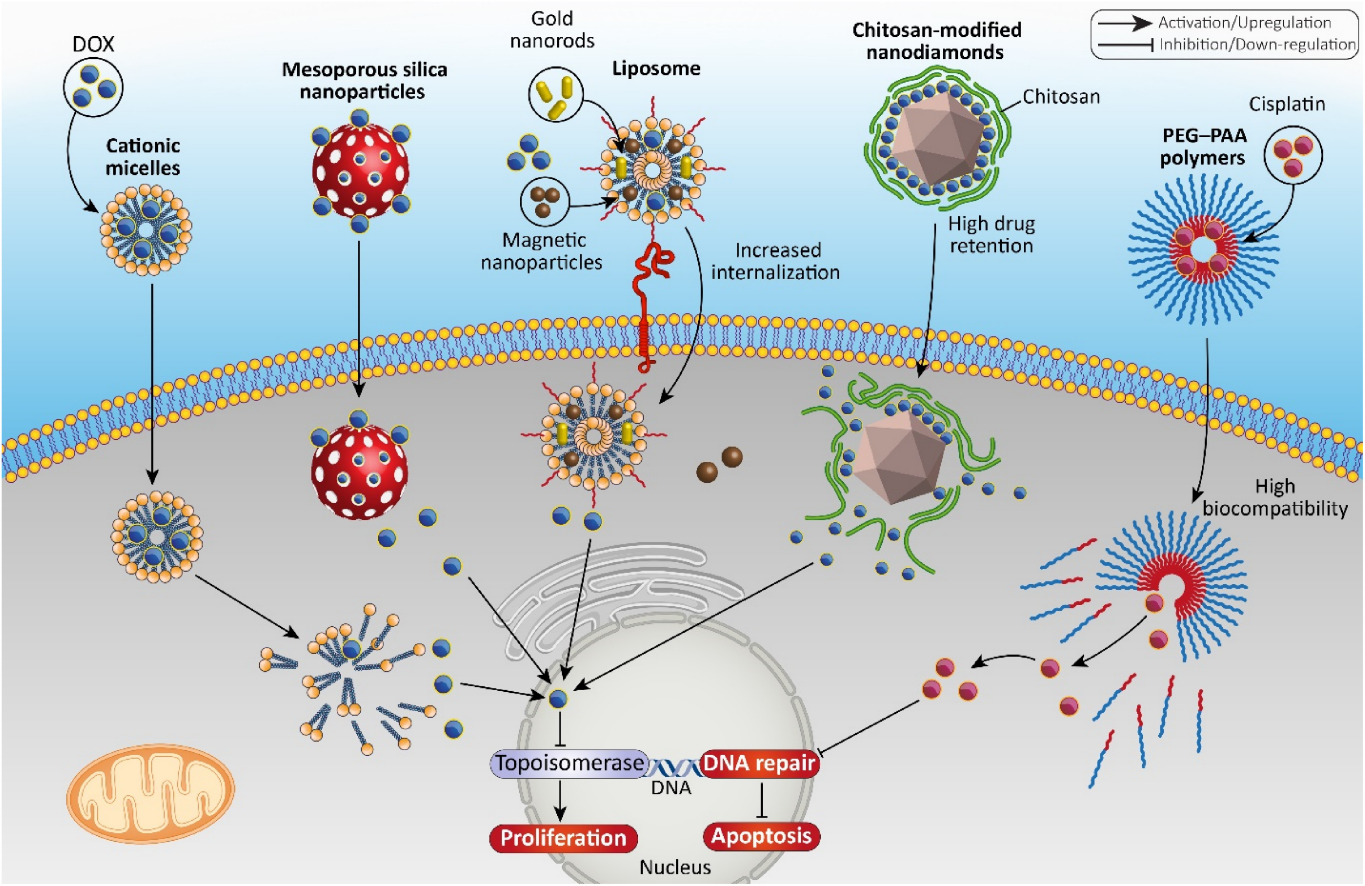
Nanomaterials for the delivery of chemotherapeutic agents in bladder cancer. The cationic micelles, mesoporous silica nanoparticles, liposomes, and chitosan nanoparticles among others, can be employed for the delivery of drugs such as cisplatin in cancer therapy to induce DNA damage and apoptosis and suppress proliferation of tumor cells. DOX, doxorubicin; PAA, poly(l‐aspartic acid sodium salt); PEG, polyethylene glycol

The previous discussions revealed the role of nano‐scale delivery systems for synthetic drugs in increasing their anti‐cancer activity. Noteworthy, nanoparticles can also increase bioavailability and therapeutic index of phytochemicals in bladder cancer therapy. The natural products have benefits such as low cost, multi‐targeting capacity, and high safety profile that have made them appropriate options in cancer therapy.^76^, ^77^ However, accumulation of natural products in bladder tumor cells is low, urging scientists to find novel methods for their delivery. PLA‐based nanostructures were prepared using precipitation technique and loaded with glycoalkaloidic extract (AE). The nanoparticles displayed a particle size of 200 nm, zeta potential of −12 and −7 mV, and encapsulation efficiency of 85%–90%. These AE‐loaded nanostructures suppressed bladder cancer progression in a concentration‐dependent manner and stimulated both apoptosis and cell cycle arrest.^78^ In another experiment, nanoemulsions were synthesized from nonionic surfactant and Neem seed oil, and then, resveratrol as a phytochemical, was loaded in these nanostructures. The nanoparticles had a particle size of 137.8 nm, and they were stable for at least 30 days. They increased accumulation of resveratrol in bladder cancer cells and significantly reduced survival and viability of tumor cells.^79^ The studies highlight the fact that delivery of phytochemicals with nanostructures is of importance in increasing their capacity for cell death induction and this is favorable for bladder cancer therapy (Figure 1).^80^, ^81^, ^82^ However, more studies regarding molecular pathways affected by phytochemical‐loaded nanostructures, their internalization route, and development of smart and other kinds of nanoparticles are vital. The advantages related to nanostructures are the evaluation of particle size, zeta potential, and encapsulation efficiency after the loading of chemotherapeutic agent. Drug‐loaded nanocarriers demonstrate low particle size (in average less than 150 nm), high stability, and good encapsulation efficiency that are of importance for purpose of chemotherapeutic agent delivery in bladder cancer treatment. Their limitation is focused on the lack of carbon‐ and metal‐based nanostructures for drug delivery in bladder cancer while most studies have focused on lipid‐ and polymeric‐based nanostructures. Furthermore, the use of nanoparticles for phytochemical delivery in bladder cancer suppression has been ignored and future studies should focus on the delivery of naturally occurring compounds with anti‐tumor activity and more importantly, their co‐delivery with synthetic chemotherapeutic agents for synergistic therapy.

## 3 NANOPLATFORMS IN GENE DELIVERY

In recent years, much attention has been directed toward the application of gene therapy for diseases, particularly cancer.^83^ Genes have more specificity compared to drugs in targeting a specific molecular pathway and therefore, they are of high importance in precision medicine.^43^ To date, various kinds of gene therapy approaches have been utilized for cancer treatment. The first kind of gene modality is the application of RNA interference (RNAi) for reducing expression level of genes. The siRNA and shRNA are two tools employed in treatment of cancer and by silencing target gene, depending on the function of genes, these tools significantly suppress proliferation and invasion of cancers. Noteworthy, siRNA and shRNA are beneficial in reversing drug resistance in tumor cells.^44^, ^84^, ^85^, ^86^, ^87^ Therefore, their application in cancer therapy can pave the way for better prognosis for cancer patients. Another approach for gene therapy is targeting noncoding RNAs (ncRNAs). These RNA molecules do not translate into proteins and they are involved in evolutionary mechanisms and regulation of biological events such as proliferation, migration, and differentiation.^88^, ^89^, ^90^ The microRNAs (miRs) are the most well‐known members of ncRNAs and they participate in pathological events. Aberrant expression of miRNAs is associated with cancer development and significant effort has been made in regulating their expression level.^91^ Although gene therapy has opened a new gate in tumor therapy, this method has faced its own problems that should be overcome. The primary obstacle in gene therapy is the low accumulation of nucleic acid drugs within tumor tissues. For instance, blood–brain barrier (BBB) prevents the entrance of agents into brain and genetic tools have poor efficacy in the treatment of brain tumors. Furthermore, the presence of blood‐tumor barrier (BTB) serves as an impediment to internalization of nucleic acid drugs to tumor cells. In vitro studies demonstrate a high efficacy of genetic tools although the application of these genes for in vivo results in their degradation in blood circulation by RNase enzymes. Therefore, encapsulation of nucleic acid drugs not only promotes their intracellular accumulation at tumor tissue, but also protects them against degradation.^44^, ^45^, ^85^, ^86^, ^87^ Therefore, current section focuses on the application of nanomaterials for genes delivery in bladder cancer therapy.

Chitosan‐hyaluronic acid dialdehyde (HAD) nanostructures can deliver Bcl‐2‐siRNA to bladder cancer site and enable the suppression of tumor growth. HAD was prepared using ethanol–water mixture and it was conjugated to chitosan nanostructures. Then, these nanoparticles provided selective delivery of siRNA to bladder cancer cells overexpressing CD44. siRNA‐loaded nanoparticles demonstrated particle size of 100–120 nm with a loading efficiency of 95% for siRNA. They had high biocompatibility and stability, and by binding to CD44 receptor on surface of bladder cancer cells, they selectively targeted tumor cells. By increasing the internalization of siRNA in bladder cancer cells, the nanostructures reduced Bcl‐2 expression to suppress viability of tumor cells.^92^ Another factor responsible for survival of bladder tumor cells is survivin. Biodegradable PLGA nanoparticles were modified with chitosan as a positively charged polysaccharide to provide their complexation with negatively charged siRNA. Compared to nonmodified nanostructures, chitosan‐modified PLGA nanoparticles demonstrated a 10‐fold increase in the internalization by bladder tumor cells. These nanostructures increased bioactivity of siRNA for up to 9 days and diminished growth rate of bladder cancer cells. In vivo experiment demonstrated 75% decrease in expression level of survivin and reduction in tumor growth.^93^

In addition to miRNAs, the level of circular RNAs (circRNAs) appears to be abnormal in bladder cancer. For example, circPRMT5 shows upregulation in bladder cancer to enhance proliferation rate. Noteworthy, siRNA can be employed with the purpose of targeting circRPMT5 in bladder cancer therapy. In an effort, geoinspired synthetic chrysotile nanotubes (SCNTs) were prepared with an outer diameter of 25 nm and a length of 100–200 nm. These nanoparticles significantly increased the internalization of circPRMT5‐siRNA by bladder tumor cells and by increasing half‐life of siRNA, they are beneficial in improving the potential of siRNA in gene silencing. The nanoparticles demonstrated high safety profile and suppressed proliferation and invasion of bladder cancer cells.^94^

Exosomes are emerging nanostructures belonging to extracellular vesicles (EVs) with sizes smaller than 100 nm and can transfer lipids, proteins, and nucleic acids among cells.^95^ Exosomes are potential therapeutic agents in bladder cancer, and they can be used for siRNA delivery in tumor suppression. As exosomes are derived from sources in body such as mesenchymal stem cells (MSCs), they have high biocompatibility and their safety profile for clinical trials has been confirmed. Exosomes derived from human embryonic kidney cells and MSCs were loaded with PLK‐1 siRNA, and compared to normal epithelial cells, high amount of exosomes was internalized in bladder tumor cells. Through increased delivery and accumulation of siRNA in tumor cells, they provided effective gene silencing and reduced progression of bladder cancer cells.^96^ Regardless of engineering exosomes for purpose of gene delivery, they can be secreted by cells in body, and by transferring, for instance, miRNA‐4792, they can enhance progression of bladder tumor cells via inducing c‐Myc signaling.^97^ In this condition, preventing biogenesis and secretion of exosomes is preferred. However, the purpose of this review is on engineered exosomes as nanostructures in gene delivery for bladder cancer therapy.

Based on these findings, application of siRNA is of importance for the suppression of proliferation and metastasis of bladder cancer cells, and their efficiency in gene silencing enhances using nanoparticles for targeted delivery.^98^, ^99^ As it was mentioned in the introduction section, chemoresistance is an increasing challenge in bladder cancer, and overcoming drug resistance requires identification of factors involved in this process and their targeting.^100^ Nrf2 is an oxidative stress regulator and is involved in reducing ROS levels via reinforcing antioxidant defense system. The overexpression of Nrf2 protein is beneficial for protection of normal cells; however, its upregulation induces resistance of tumor cells to cytotoxic impact of anti‐cancer agents.^45^ Therefore, downregulation of Nrf2 is an ideal strategy in cancer therapy. For this purpose, guanidine‐terminated carbosilane dendrimers (GCDs) were employed to deliver Nrf2‐siRNA in suppressing cisplatin resistance in bladder cancer. The nanocarriers had various particle sizes such as 346.8, 128.1, and 325.6 nm and zeta potentials of −12.10, 2.72, and 10.34 mV. The nanocarriers possessed high biocompatibility, ensuring their further application for clinical trials and treatment of bladder cancer. These nanocarriers significantly reduced expression level of Nrf2 to induce apoptosis and oxidative stress in bladder cancer cells, leading to a remarkable decrease in proliferation and invasion. Furthermore, Nrf2‐siRNA‐loaded GCDs promoted sensitivity of bladder cancer cells to cisplatin chemotherapy.^101^ Therefore, various aspects of bladder cancer cells including growth, metastasis, and therapy response are tightly regulated by siRNA‐loaded nanocarriers.

KDM6A is a factor responsible for regulating progression of bladder tumor cells. KDM6A mutation in bladder cancer is associated with immune escape of tumor cells and reduces infiltration of immune cells.^102^ There is a close association between KDM6A and bladder cancer invasion. At molecular level, KDM6A promotes the expression level of ARHGDIB to downregulate Rac1 in suppressing metastasis of bladder cancer cells.^103^ An experiment has focused on delivery of KDM6A‐mRNA by mucoadhesive nanostructures in bladder cancer therapy. The use of nanostructures enabled enhanced exposure of KDM6A‐mRNA to bladder cancer site and provided sustained delivery. By promoting KDM6A expression, a significant decrease occurred in metastasis of bladder tumor cells. Furthermore, mRNA‐loaded nanostructures showed a synergistic impact with other clinically approved drugs.^104^

In addition to RNAi and noncoding RNAs, studies have focused on the application of CRISPR/Cas system as a powerful genetic tool in bladder cancer therapy. The CRISPR/Cas system is beneficial in the identification and therapeutic targeting of factors involved in the progression of bladder cancer cells. For instance, whole‐genome screening using CRISPR system demonstrated the role of MSH2 overexpression in triggering cisplatin resistance in bladder cancer.^105^ The CRISPR/Cas9 and Cas13 are the most common types of CRISPR system employed for the treatment of bladder cancer. The CRISPR system can reduce expression levels of SMAD7e and CacyBP, among others to suppress progression of bladder cancer cells.^52^, ^106^, ^107^, ^108^, ^109^ To date, only one experiment has used nano‐scale delivery systems for CRISPR/Cas13a in bladder cancer therapy. The hydration method was used for the synthesis of liposomes and then, CRISPR/Cas13a was loaded to target hVEGFR2 that showing upregulation in bladder tumor cells. The nanostructures had a particle size of 855 nm with a zeta potential of 25.7 mV. This delivery system effectively downregulated the expression level of *EGFP* gene to suppress the progression of bladder tumor cells.^110^

**FIGURE 2:**
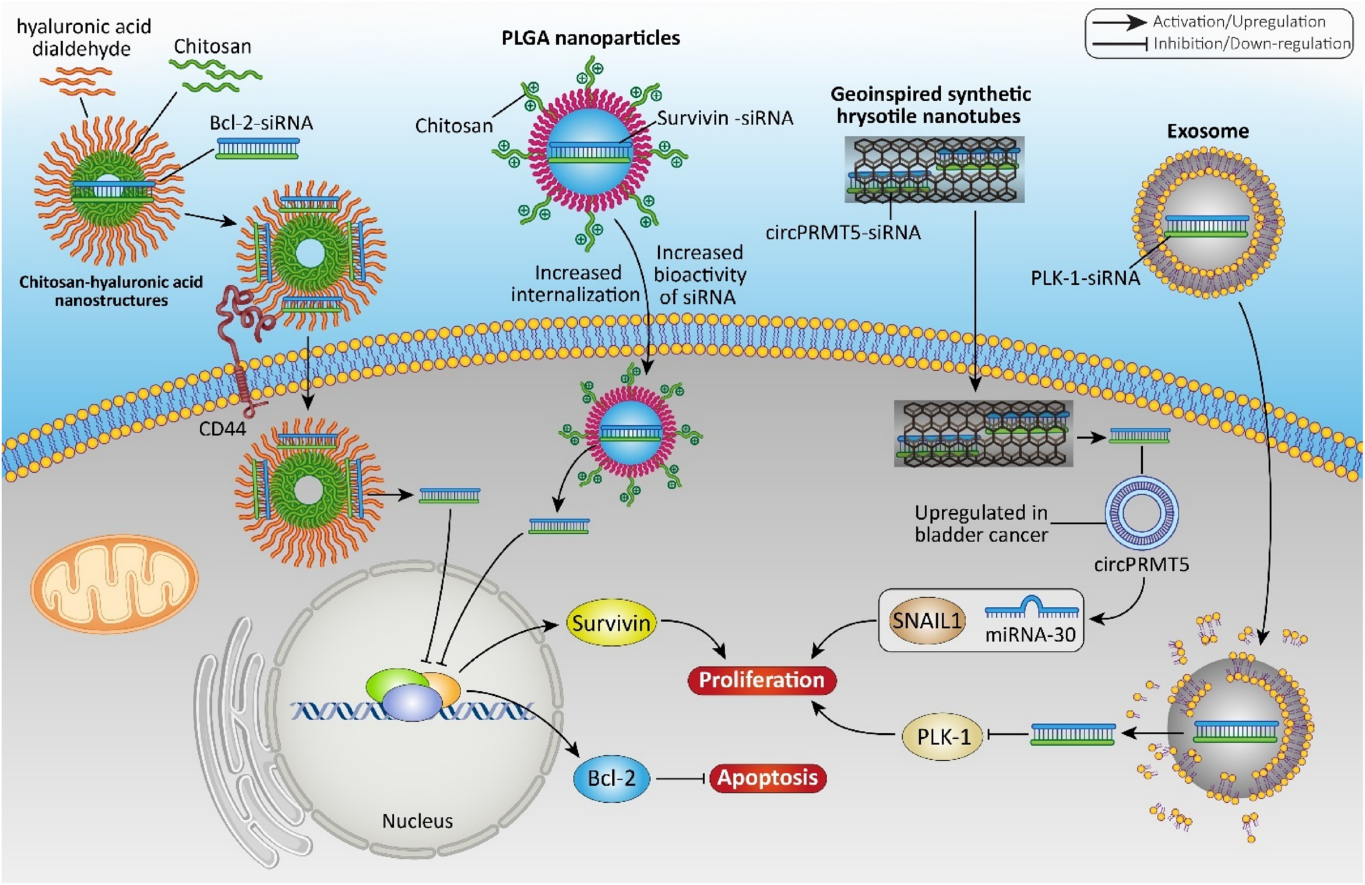
The nanomaterials for gene delivery in bladder cancer. siRNA is the most well‐known gene loaded on nanoparticles for bladder cancer therapy. miRNAs can also be used in bladder cancer therapy and the final aim is to induce apoptosis and to suppress proliferation. PLGA, poly d,l‐lactic‐co‐glycolic acid; PLK‐1, polo‐like kinase 1; siRNA, small interfering RNA

Although studies have yielded promising results with regard to the application of nanostructures for gene therapy in bladder cancer, there are some issues and limitations. The first is that metal‐ and carbon‐based nanocarriers have not been used for gene delivery in bladder cancer. Most studies have concentrated on polymeric nanoparticles. Another limitation is that most studies are dealing with siRNA delivery. However, delivery of miRNAs, shRNA, and CRISPR/Cas9 system in bladder cancer therapy has been somewhat ignored and more studies should be performed to show how their targeted delivery can boost their anti‐cancer activity. As genes have a negative charge, their stable complexation is performed with positively charged nanocarriers. Therefore, in addition to chitosan, other kinds of polymers with positive charge are examined in terms of complexation with genes in bladder cancer therapy. Figure 2 provides a summary nanocarriers used for gene delivery in bladder tumor treatment.

## 4 NANOPLATFORMS IN CO‐DELIVERY

The delivery of genetic tools promotes their efficacy in the regulation of gene expression, and delivery of anti‐cancer agents increases their tumor‐suppressor activity. Various kinds of nanostructures including polymeric‐, lipid‐, metal‐, and carbon‐based nanomaterials have been employed for co‐delivery of genes and drugs to exert a synergistic impact on the suppression of cancer progression.^85^ A similar phenomenon has been applied to bladder cancer. Calcium carbonate (CC) nanostructures were modified with cancer cell membrane followed by loading miRNA‐451 and Adriamycin. The resulting nanocarriers demonstrated particle size of 100 nm and they effectively suppressed the progression of bladder cancer. By delivering miRNA‐451, the nanocarriers significantly reduced the expression level of P‐gp to prevent drug efflux and increase internalization of Adriamycin in bladder cancer cells, resulting in tumor suppression in vitro and in vivo.^111^ However, only one experiment has evaluated the potential of nanoplatforms for co‐delivery of genes and drugs in bladder cancer. Therefore, more evidence is needed to show how the combination of miRNAs with other anti‐cancer agents such as CP and PTX can improve their efficacy in bladder cancer treatment. Furthermore, as siRNA is a beneficial mainstay in bladder cancer suppression,^112^, ^113^, ^114^, ^115^ studies are warranted to exhibit co‐delivery of siRNA and drugs in combination with cancer therapy. Furthermore, as genetic tools have negative charge and anti‐cancer drugs can be hydrophilic or hydrophobic, experiments should show how modification of nanoparticles with various polymers can result in the formation of a stable complex for co‐delivery of both genes and drugs (Table 1).

**TABLE 1:** Nanoplatforms for the delivery of chemotherapeutic agents in bladder cancer therapy

Nanovehicle	Particle size (nm), Zeta potential (mV)	Cargo	Remarks	References
Mesoporous silica nanoparticles	170.2 nm, −15.9 mV	Doxorubicin	Increased cellular uptake due to peptide modification and binding to receptors on the surface of bladder tumor cells Suppression of tumor growth in vitro and in vivo	46
Mesoporous silica nanoparticles	75.5 nm, +33.5 mV	Doxorubicin	The functionalization of nanoparticles with thiol enhances their mucoadhesive features Prolonged drug release	116
Poly(amidoamine)‐modified mesoporous silica nanoparticles	119.4, 127.7, and 124.5 nm	Doxorubicin	Encapsulation efficiency up to 95.5% High mucoadhesive activity Prolonged and pH‐responsive release of DOX	117
RGD‐Fe_3_O_4_/CaP/Alg nanoparticles	200 nm, −22.9 mV	Doxorubicin	High biocompatibility and ease of surface functionalization High cellular uptake, pH‐sensitive release of drug and increasing efficacy of chemotherapy in bladder tumor suppression	118
PAMAM dendrimer nanoparticles	13 nm, +2.78 mV	Doxorubicin	High safety profile and low particle size Increase in DOX accumulation at tumor site Reduction in adverse impacts of DOX on normal cells Suppression of tumor growth in animal model	119
rHDL/AD‐32 nanoparticles	18 and 20 nm	Valrubicin	Decreased IC_50_ value of cytotoxic drug Receptor‐mediated uptake in bladder tumor cells High stability and promising results as drug delivery systems	120
Polymeric nanostructures	281 nm, −34.3 mV	Cisplatin	Mucoadhesive property Stability at room temperature 30% cisplatin release during the first 4 h and prolonged release for 4 days Suppressing bladder tumor progression via reducing IC_50_ of cisplatin	68

## 5 NANOPLATFORMS IN PHOTOTHERAPY

The red‐light irradiation was first used in 1975 in killing tumor cells. Since then, photodynamic therapy (PDT) has been considered as a promising strategy in the treatment of cancer.^121^ Three main components are required for an appropriate PDT including photoactive drug, light with a proper wavelength, and molecular oxygen.^121^ Upon exposure to light, excitation of photoactive drug to its triplet state occurs that interacts with oxygen in tissue, leading to generation of reactive oxygen species (ROS). PDT is considered as one of the most promising approaches in treatment of cancer and it has low side effects and partially affects neighboring cells and tissues.^122^ Photothermal therapy (PTT) is also another approach for minimally‐invasive treatment of cancer that uses a photothermal agent for converting light to energy to eliminate tumor cells.^123^ Noteworthy, both PDT and PTT can be co‐utilized to suppress progression and viability of tumor cells in a synergistic manner.^124^, ^125^, ^126^, ^127^ The current section focuses on the application of PDT and PTT in the treatment of bladder cancer.

**FIGURE 3:**
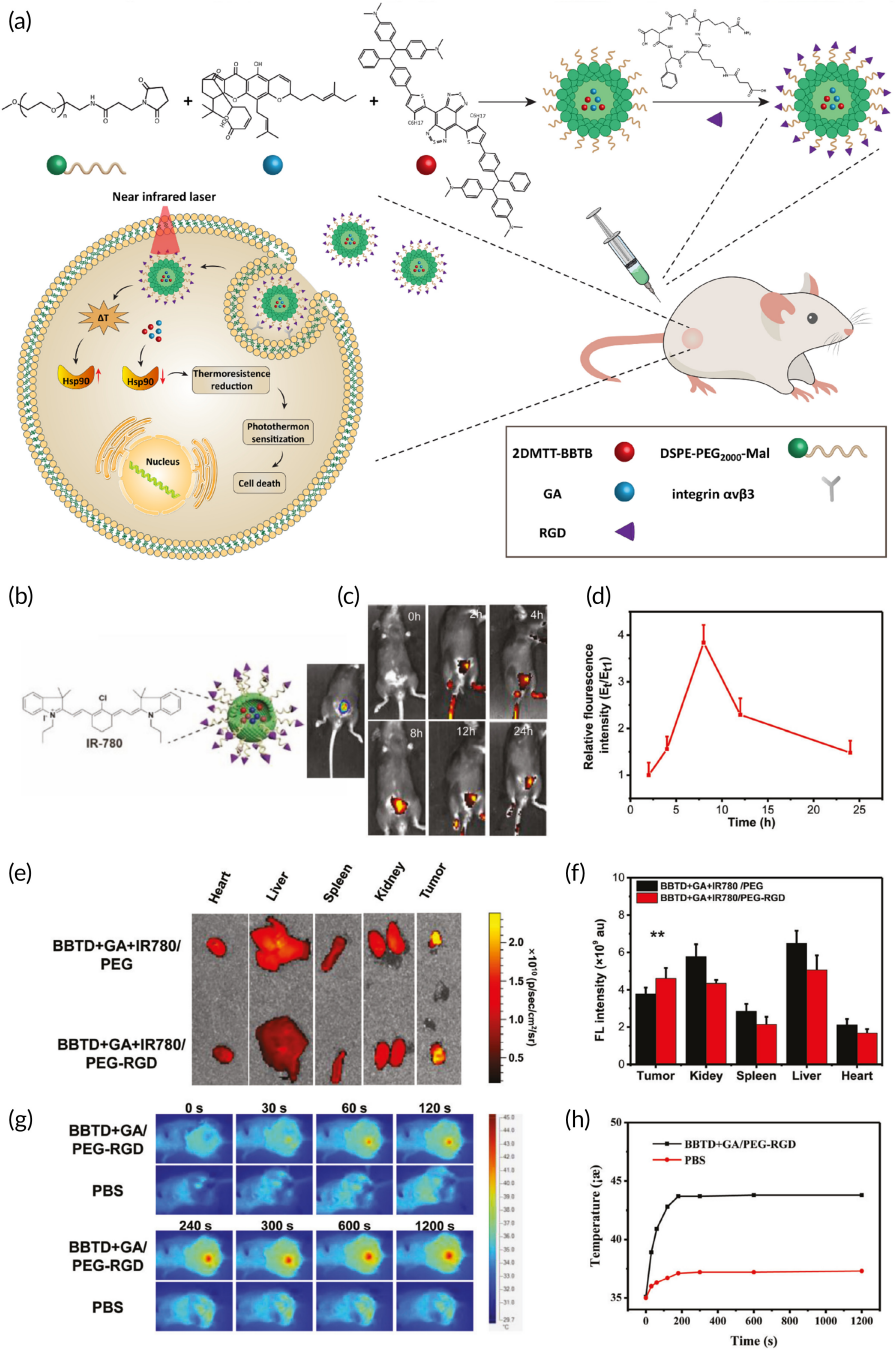
(a) Injection of nanomaterials for PDT and suppressing bladder cancer progression. (b) Schematic illustration of fluorescent BBTD+GA + IR780/PEG‐RGD nanoparticles and chemical structure of IR780. (c) In vivo fluorescence imaging of tumor after intravenous injection of BBTD+GA + IR780/PEG‐RGD nanoparticles. (d) Tumor fluorescence changes after intravenous injection of BBTD+GA + IR780/PEG‐RGD nanoparticles. (e) Ex vivo fluorescence images of different organs and tumors. F) Fluorescence intensities of different organs. (g) Infra‐red thermal images of whole mice bodies after 808 nm laser irradiation (0.8 W cm^−2^, 20 min). (h) Tumor temperature evaluation as a function of 808 nm laser irradiation time. Reprinted with permission from Zeng et al.^129^ from Wiley

Currently, surgery is not considered as a promising option in bladder cancer treatment due to tumor recurrence and metastasis, and its combination with chemotherapy has other problems such as side effects. Therefore, innovative approaches such as PTT are employed for the treatment of bladder cancer. Fe(III)‐doped polyaminopyrrole (FePPy‐NH_2_) nanostructures with high safety profile and high half‐life in blood circulation of as much as 7.59 h were employed in bladder cancer therapy. The nanoparticles preferentially targeted bladder tumor cells with an accumulation rate of 5.07 %ID/g. After accumulation at bladder tumor site, near‐infrared (NIR) light was employed to provide bladder tumor ablation with photothermal conversion efficiency of 44% and lack of recurrence.^128^ The efficiency of PTT in bladder cancer ablation depends on the conversion efficiency of synthesized nanomaterials. In a recent study for improving the capacity of nanostructures in heat generation for treatment of MIBC, nonradiative heat production was boosted via introducing twisted intramolecular charge transfer (TICT) and molecular motions. This work designed a novel molecule known as 2DMTT‐BBTD in which tetraphenylethenes functioned as molecular rotors, long alkyl chain grafted thiophene mediated TICT state formation, and maintained molecular motions in aggregate, and on the other hand, electron‐withdrawing BBTD unit was beneficial in increasing TICT effect. The 2DMTT‐BBTD had the capacity of NIR irradiation absorption and PCE value of 74.8% under 808 nm wavelength. In addition to 2DMTT‐BBTD, gambogic acid (GA) as an inhibitor of heat shock protein 90 (HSP90) was loaded into nanostructures to prevent cancer cell thermotolerance. The accumulation of nanostructures in tumor tissue was boosted via surface modification with RGD peptide. These nanoparticles could provide selective and effective PTT in MIBC with minimal impact on neighboring tissues to suppress bladder tumor progression (Figure 3).^129^ Overall, surface modification of nanocarriers with ligands such as phosphatidylserine that can interact with receptors on surface of bladder cancer cells is a promising strategy for increasing the accumulation of nanoparticles in tumor site and potentiating hyperthermia.^130^ This surface modification and its impact on internalization will be discussed more in the next sections.

Another study reported self‐assembled polymeric nanomaterials containing IR‐780 for PTT in bladder cancer. The nanostructures were modified with hyaluronic acid (HA) to selectively target bladder tumor cells overexpressing CD44 receptor. The nanoparticles had a particle size of 171.3 nm and were degraded at bladder tumor site by hyaluronidase. These nanoparticles demonstrated high photothermal conversion efficiency, and after application of 2.5, 5, 10, and 20 μg/ml of nanostructures, temperature increases were recorded up to 11.2, 18.6, 26.8, and 32.3°C, respectively. Upon 808 nm laser irradiation, viability of MB‐49 cells diminished and in vivo experiment revealed the accumulation of nanostructures in bladder tumor tissue with partial internalization in a healthy bladder wall. After irradiation, tumor tissues demonstrated a temperature of 48.1°C and reduced tumor volume, confirming potential of HA‐IR‐780 polymeric nanostructures in bladder tumor ablation.^131^ Although most of the experiments focused on PTT by nanoparticles in bladder tumor, there are studies showing that PDT is also beneficial in bladder cancer ablation.

Intravenous administration of photosensitizers has been performed for the treatment of bladder cancer, however, poor bioavailability of photosensitizers and chance of systemic phototoxicity has led to the development of novel methods for photosensitizer application. A novel and innovative method, in this case, is intravesical instillation‐based PDT. Nonetheless, it suffers from drawbacks including poor transmucosal efficiency, hypoxia regulation deficiency, and low safety profile. Therefore, human serum albumin (HSA) nanoparticles modified with fluorinated chitosan were recently developed for co‐loading of nitazoxanide (NTZ) as antiparasitic agent and chlorin 36 (Ce6) and to improve biocompatibility of this transmucosal barrier. Compared to nonmodified nanoparticles, the chitosan‐modified HSA nanoparticles demonstrated higher cellular uptake and biocompatibility. The Ce6 provides PDT and NTZ by modulating metabolism of bladder tumor cells, and potentiates PDT for tumor suppression.^92^ The porphyrins are other agents that can be employed as photosensitizers, because they are robust, have the capacity of singlet oxygen generation and possess great chemical, photo, thermal, and oxidative stability.^132^, ^133^, ^134^, ^135^ However, poor accumulation of porphyrins at tumor site significantly diminishes their potential for being used as photosensitizer. A recent experiment synthesized hybrid nanocarriers composed of lipid nanoparticles coated with porphyrin‐chitosan with particle size of 130 nm and zeta potential of 27.1 mV. Although conjugation of porphyrin‐chitosan to lipid nanocarriers enhanced their particle size, this combination was beneficial in improving stability of these carriers and mediated their self‐assembly and spherical shape formation. These nanostructures demonstrated mucoadhesive property and increased internalization of porphyrin, leading to PDT and bladder tumor suppression.^136^

**FIGURE 4:**
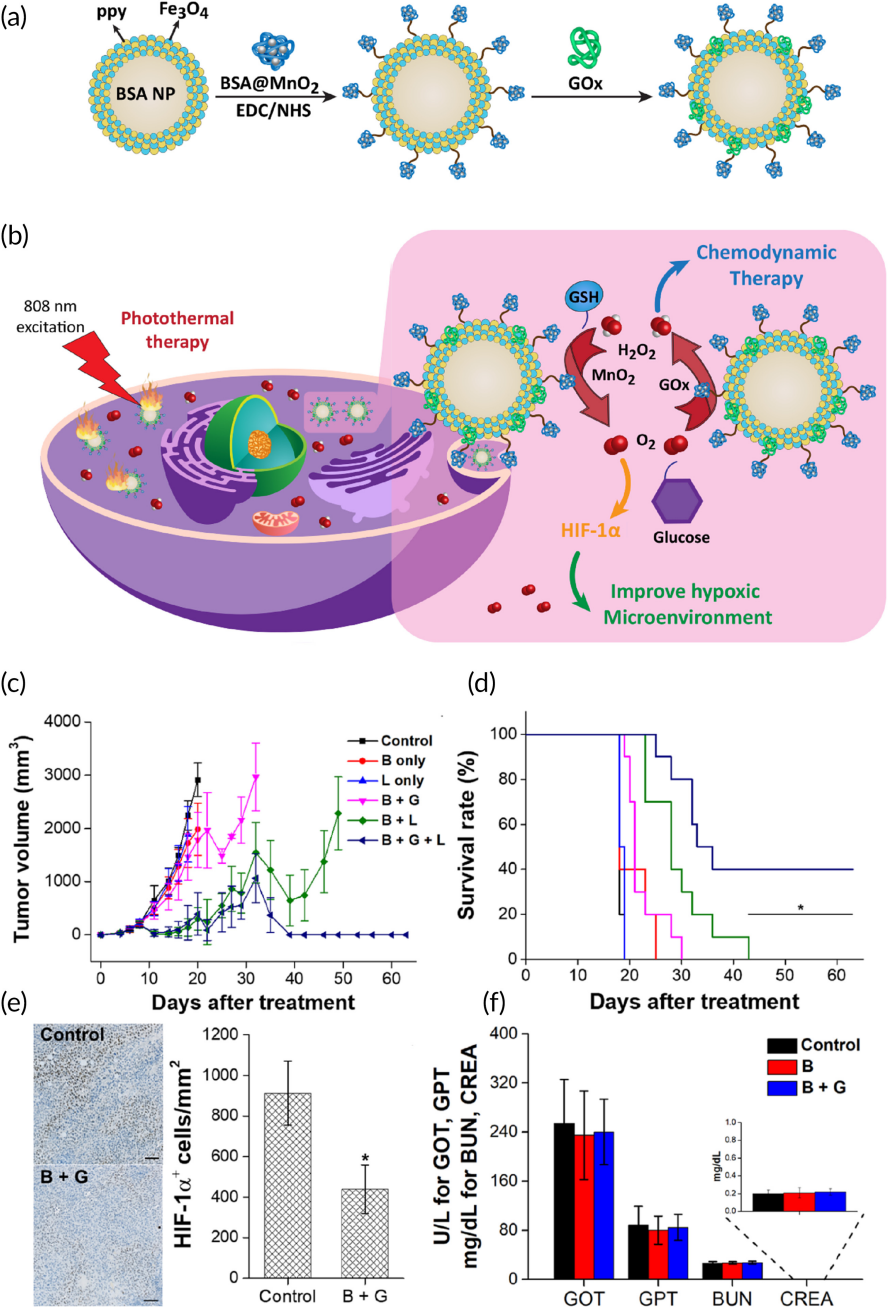
(a) Schematic illustration for the preparation of GOx@MBSA‐PPy‐MnO_2_ NPs. (b) Their application in efficient PTT/Glucose‐Triggered CDT and GSH‐Triggered hypoxia relief of bladder cancer, (c) tumor weight, (d) survival rate, (e) expression level of HIF‐1α, and (f) hepatic function. Reprinted with permission from Chen et al.^108^ from ACS

These studies demonstrated that PDT and PTT are beneficial in treatment of bladder cancer. Notably, phototherapy can be co‐applied with chemotherapy and immunotherapy in synergistic inhibition of bladder cancer. Similar to drug resistance, tumor cells have capacity of resistance to PTT via overexpression of heat shock proteins. A recent study reported nanocarriers for combining PTT, glucose‐induced chemodynamic therapy (CDT), and glutathione (GSH)‐mediated hypoxia relief. The GOx@MBSA‐PPy‐MnO_2_ nanostructures demonstrated high photothermal conversion efficiency via release of Mn^2+^ and enhanced transformation of H_2_H_2_ into singlet oxygen and hydroxy radicals by Fenton‐like reaction to suppress bladder tumor progression and reduced hypoxia. Noteworthy, GOx induced catalysis of glucose to enhance the generation of H_2_O_2_ for CDT. The in vitro and in vivo experiments showed that such a combination is of importance to prevent bladder cancer progression and PTT resistance (Figure 4).^108^

One of the important progresses in field of bladder cancer therapy is development of image‐guided PTT. Such procedure allows to understand accumulation of nanomaterials at site and localization of PTT at tumor to prevent damage to normal and healthy tissues. For this purpose, Fe(III)‐doped polyaminopyrrole (FePPy‐NH_2_) have been designed with high biocompatibility and half‐life of 7.59 h. Noteworthy, the prepared nanostructures demonstrated high accumulation at bladder cancer site (5.07%ID/g). The underlying reason for the coordination of Fe(III) and amino group in the structures of these nanoparticles is to provide both magnetic imaging and PTT. The nanoparticles had photothermal conversion efficiency of 44.3% and by inducing PTT, provided ablation of tumor progression, while they provided imaging of bladder tumor site simultaneously.^128^

**FIGURE 5:**
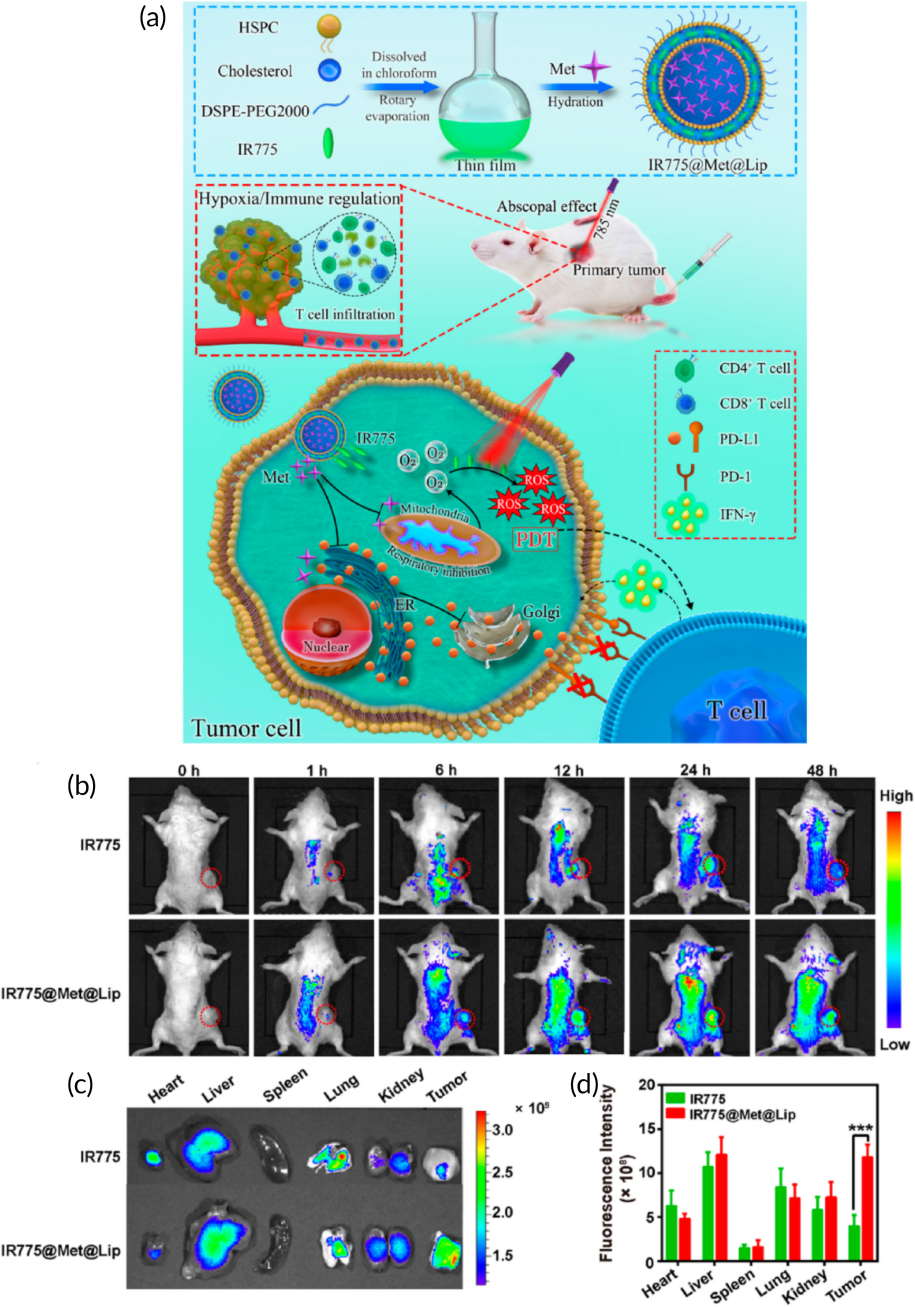
(a) Synthesis of nanomaterials and their application in PDT immunotherapy. (b) Real‐time NIR fluorescence images of tumor‐bearing mice after intravenous injection of IR775 or IR775@Met@Lip at different time points. Dotted red circle represents the tumor. (c) Ex vivo fluorescence images of mice at 24 h after indicated treatments. (d) Quantification of IR775 fluorescence signal in the tumor tissues and main organs at 24 h after indicated treatments. Reprinted with permission from Xiong et al.^140^ from ACS

Single‐walled carbon nanotubes (SWCNTs) have been employed for DOX delivery and providing PTT. The drug loading efficiency of SWCNTs was 40% and they had a particle size of 220 nm. These nanostructures significantly accumulated at tumor site, and by providing chemotherapy and PTT, they suppressed the progression of bladder cancer.^137^ Hence, combination of chemotherapy and phototherapy shows some promising results in bladder cancer therapy.^138^ This benefit has been confirmed in both in vitro *and* in vivo studies.^139^ Another issue worth mentioning for the application of phototherapy is its impact on immune system. It has been reported that PDT has the capacity of ROS overgeneration and enhancing anti‐tumor immunity via T cell infiltration. However, PDT can enhance PD‐L1 expression to mediate immunosuppression. Liposomes were used to co‐deliver metformin and IR‐775 to enhance ROS overgeneration and down‐regulate PD‐L1 expression to mediate PDT and immunotherapy in bladder cancer (Figure 5).^140^ Therefore, another approach is combination of phototherapy and immunotherapy in suppressing bladder tumor progression (Table 2). Several conclusions can be drawn from these discussions that both PDT and PTT demonstrate high potential in triggering cell death in bladder tumor. The application of PDT or PTT along with chemotherapy and immunotherapy can lead to synergistic therapy of bladder cancer. Furthermore, drug resistance of bladder tumor cells can be suppressed using nanoarchitecture‐mediated PDT or PTT. Notably, due to capacity of bladder cancer cells in developing resistance to phototherapy, the application of heat shock inhibitors is suggested.

**TABLE 2:** Nanostructures for the purpose of phototherapy in bladder cancer

Nanovehicle	Particle size (nm), Zeta potential (mV)	PTT or PDT	Remarks	References
Fe(III)‐doped polyaminopyrrole nanoparticles	100 nm	PTT	High biocompatibility Blood circulation time of 7.59 h High accumulation rate in tumor site Conversion efficiency of 44.3%, reducing tumor progression, and preventing recurrence	128
GOx@MBSA‐PPy‐MnO_2_ nanoparticles	80.3, 94.7, and 98.3 nm, −11.4 mV	PTT	Reducing proliferation rate Providing simultaneous chemotherapy and phototherapy	108
Fe_3_O_4_@PDA‐VCR‐FA superparticles	128.57 and 133.87 nm, −35.2 mV 19.3%	PTT	Inducing PTT effect High biocompatibility and blood circulation time High retention in tumor site	141
Hyaluronic acid‐IR780 nanoparticles	171.3 nm	PTT	Partial accumulation at normal bladder wall and high and preferential accumulation in bladder tumor tissue for PTT Targeting bladder cancer cells overexpressing CD44	131
Cu‐Fe‐Se nanosheets	70 nm	PTT	High photothermal conversion efficiency of 78.9% Biodistribution in bladder that can be used for PTT and chemotherapy	142
Multi‐responsive liposomes	230 nm	PTT	High drug loading efficiency and capacity of 95% release of DOX after 3 h Increased cellular uptake via binding to folate receptors on cell surface Providing both PTT and chemotherapy	49
Polydopamine nanoparticles	244.2 nm, 35.3, and 31.2 mV	PDT and PTT	High colloidal stability and release of photosensitizer until day 5 Increased cellular uptake via endocytosis Providing both PDT and PTT in bladder cancer suppression	138
Poly (OEGMA)‐PTX@Ce6 nanostructures	168.2 nm	PDT	High cellular uptake and suppressing bladder tumor progression up to 98% Increasing ROS generation to induce cell death and to reduce viability of bladder tumor cells Apoptosis induction Downregulation of TGF‐β and TNF‐α	139

## 6 NANOPLATFORMS IN IMMUNOTHERAPY

In recent years, treatment of cancer by modifying immune system has gained much attention and more efforts have been dedicated worldwide toward regulating the immune status in the field of oncology. The immunotherapy uses immune system of a patient in favor of preventing development of cancer and reinforcing the defense mechanism.^143^ The first report of using immunotherapy in clinical course returns to 1890s, when William Coley used a bacterial toxin, and although clinical result of this study was small, it provided new insight towards using immune system alteration in cancer therapy.^144^ For a long time, immunotherapy has been considered as a promising option in treatment of bladder cancer.^145^ The most well‐known agent employed for immunotherapy in bladder cancer is Bacillus Calmette–Guérin (BCG) that is a live attenuated form of *Mycobacterium bovis* and is capable of stimulating immune response via enhancing levels of cytokines and interferon‐gamma to inhibit progression of bladder cancer.^146^, ^147^ The checkpoint inhibitors including atezolizumab, pembrolizumab, avelumab, and nivolumab, among others, are extensively utilized for immunotherapy in bladder cancer.^145^ Although these checkpoint inhibitors have provided some promising results in the treatment of bladder cancer and different experiments are currently being performed in clinical trials, prognosis of bladder cancer patients is still undesirable, and more intense effort is needed. Cancer cells induce PD‐L1/PD‐1 axis to provide immune escape,^148^, ^149^ therefore, novel strategies for bladder cancer immunotherapy are required.

As it was mentioned, BCG can be used as an immunotherapeutic agent in bladder cancer therapy. However, application of BCG can cause local and systemic toxicity, and these unfavorable reactions and irritative symptoms have urged scientists to focus on the targeted delivery of BCG. Cationic chitosan nanoparticles were employed for intravesical delivery of BCG and they demonstrated particle size of 269–375 nm and encapsulation efficiency of 42%. The positive zeta potential of nanoparticles makes them a good nanocarrier for the intravesical delivery of BCG. The BCG‐loaded chitosan nanoarchitectures had high biocompatibility and led to no systemic adverse impact after application. These nanostructures accumulated in bladder cancer tissue and significantly improved the survival rate of the cancer models.^150^

**FIGURE 6:**
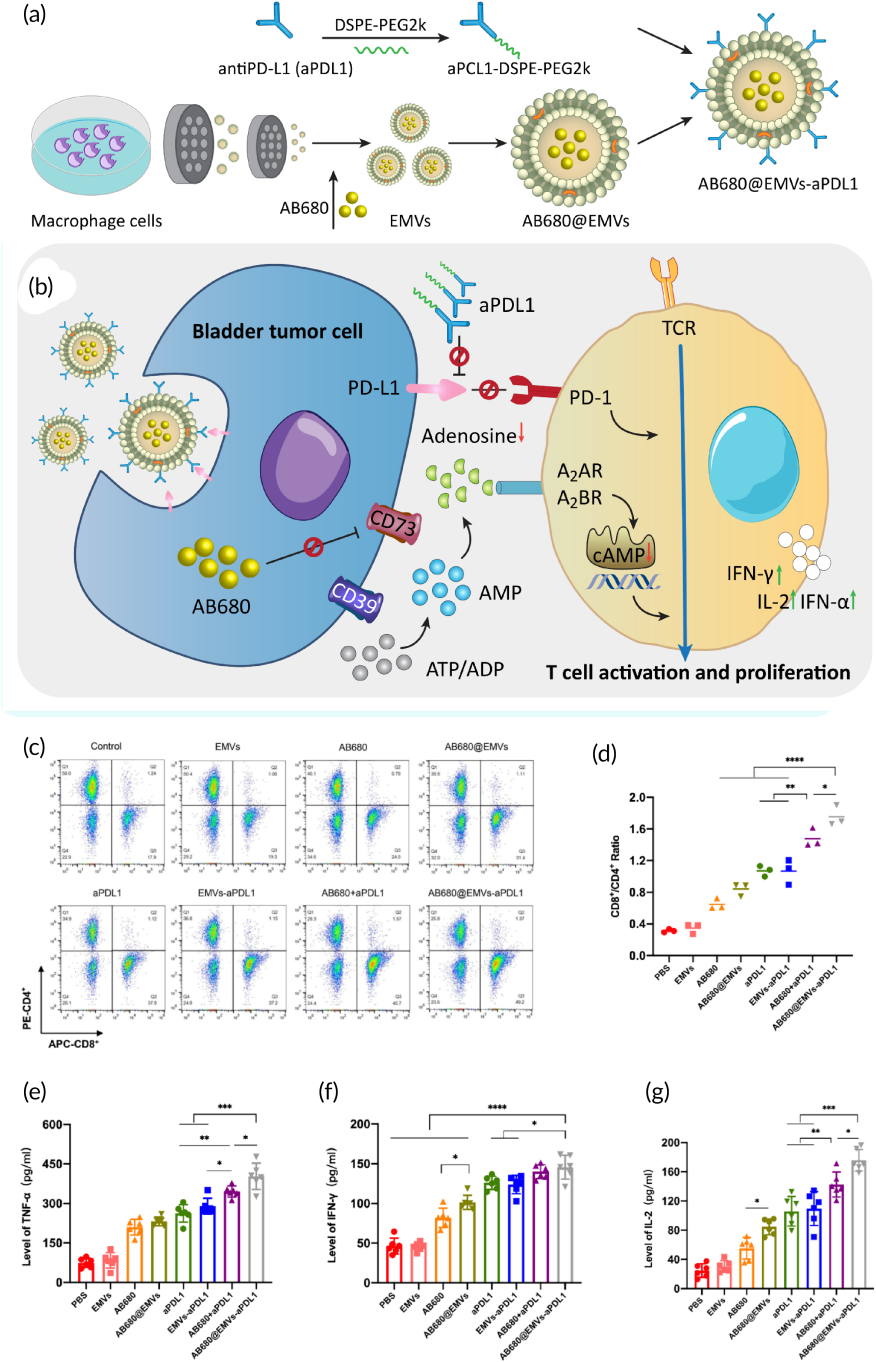
(a,b) Preparation of nanocarriers and their mechanism of action in suppressing bladder cancer progression. (c) Flow cytometry showing the CD8^+^/CD4^+^ T‐cell ratio, D) CD8^+^/CD4^+^ ratio after immune stimulation in all groups (*n* = 3). (e–g) TNF‐α, IFN‐γ, and IL‐2 concentrations in the tumor tissues from the various groups. Reprinted with permission from Zhou et al.^153^ from ACS

Overexpression of PD‐L1 is in favor of immune escape in bladder cancer. As an upstream mediator, FGFR3 decreases expression level of PD‐L1 via mediating its degradation to increase T cell‐mediated anti‐tumor immunity in reducing progression of bladder tumor cells.^151^ On the other hand, CALD1 elevates expression level of PD‐L1 through JAK/STAT signaling induction to mediate immune escape in bladder cancer.^152^ Therefore, immune escape is a complex process in bladder cancer and various mechanisms are involved in this process in which PD‐L1 plays a central role. The macrophage‐derived exosome‐mimetic nanovesicles (EMVs) have been utilized for delivery of CD73 inhibitor (AB680) and PD‐L1 antibody. The nanoparticles demonstrated a particle size of 218.2 nm with a zeta potential of −13.7 mV. In vitro and in vivo experiments demonstrated high safety profile, good stability, and targeted delivery of nanoparticles to bladder cancer. The PD‐L1 downregulation prevented immune escape and CD73 inhibitor diminished the generation of extracellular adenosine. This combination impact significantly elevated the infiltration of cytotoxic T lymphocytes in bladder cancer to augment tumor immunotherapy (Figure 6).^153^

The penetration of nanoparticles into bladder cancer tissue is of importance for immunotherapy. Furthermore, synergistic impact by delivery of chemotherapeutic agents and immune system‐related factors can be performed to provide chemoimmunotherapy. Fluorinated chitosan has the ability of opening tight junctions in bladder epithelium and it can be used for cargo delivery. Fluorinated chitosan can be modified with PEG and glutaraldehyde to self‐assemble into nanostructures. At bladder tumor site, there are collagen amines that aldehyde groups on the surface of nanostructures can adhere to them. Then, fluorinated chitosan opens tight junctions and promotes penetration into bladder tumor tissue. Pirabucin as chemotherapeutic agent and IL‐12 as immune stimulator were loaded and led to chemoimmunotherapy in bladder cancer (in vitro and in vivo).^154^

**FIGURE 7:**
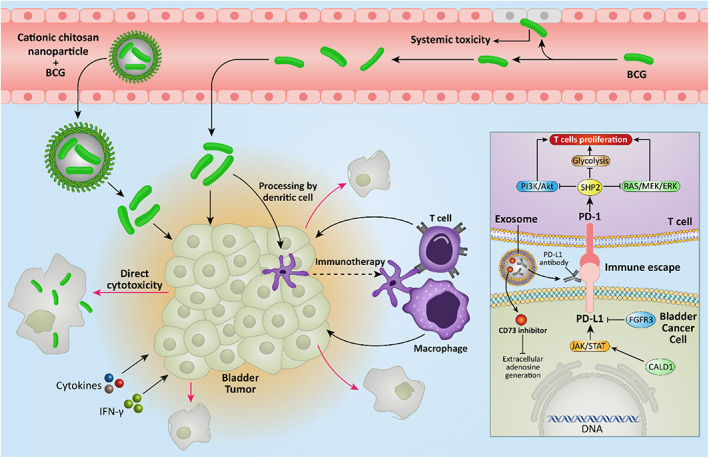
Nanoparticles and immunotherapy in bladder cancer. The tumor microenvironment is comprised of various cells including tumor cells, T cells, macrophages, normal cells, and cancer cells. Nanoparticles with high biocompatibility can circulate in bloodstream to reach to tumor site. Then, cargo‐loaded nanoparticles can mediate tumor microenvironment remodeling and regulate molecular pathways related to immune system to suppress immune evasion of cancer cells

Although studies have revealed an evident role of nanoparticles in bladder cancer immunotherapy, there are still some gaps to be evaluated in future experiments. The tumor microenvironment (TME) consists of various kinds of cells including T cells, immune cells, normal cells, and cancer cells. Previous research has only focused on T cells and how they can be regulated by nanostructures in bladder cancer immunotherapy, while their interaction with other cells has been ignored. Furthermore, the polarization of macrophages (M1 and M2) is of importance and M2 polarized macrophages demonstrate immunosuppressive activity.^155^, ^156^, ^157^, ^158^ Therefore, future studies should also focus on targeting macrophages by nanocarriers in bladder tumor immunotherapy (Figure 7).

## 7 STIMULI‐RESPONSIVE NANOPLATFORMS

**FIGURE 8:**
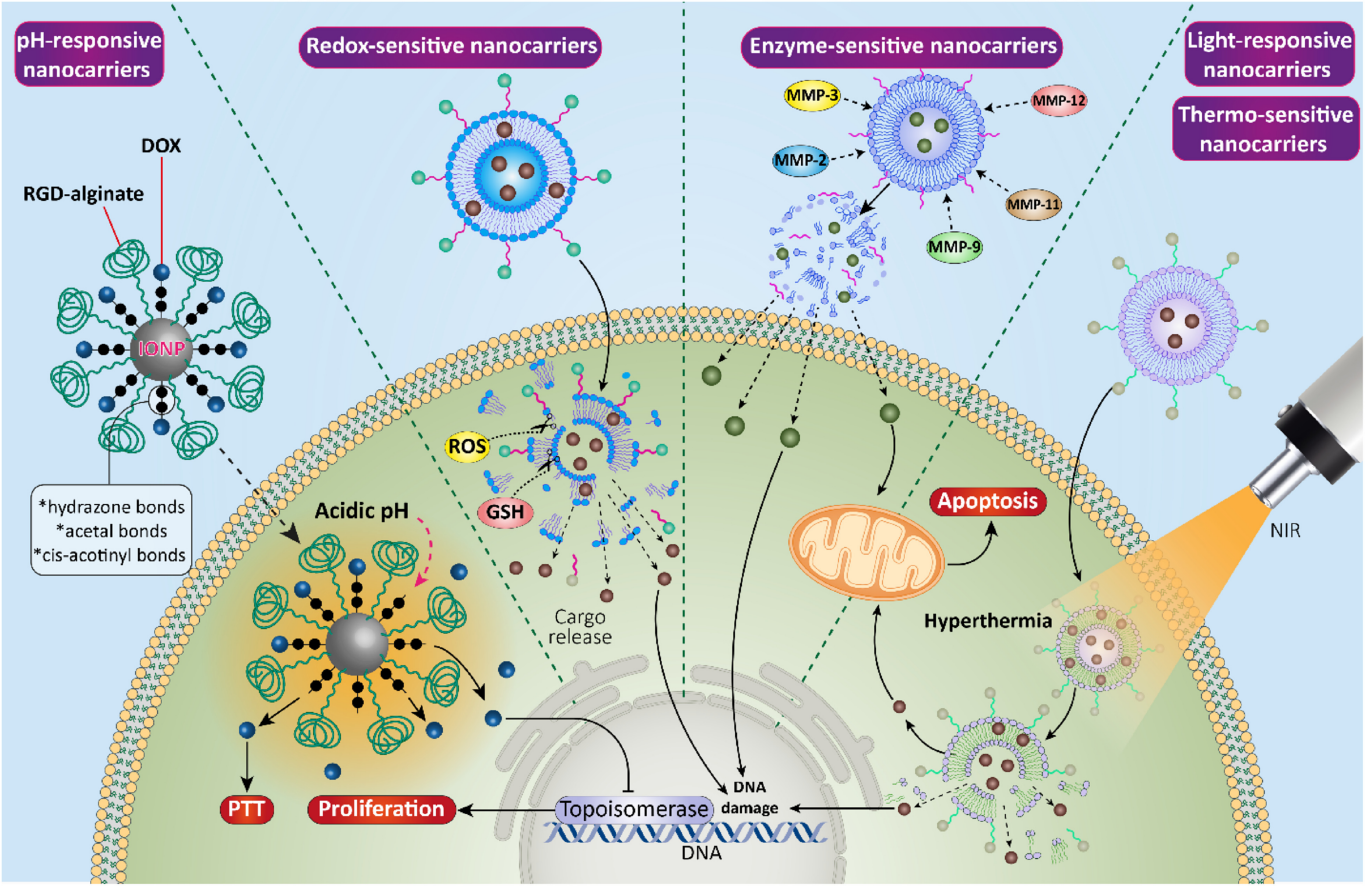
A schematic representation of stimuli‐responsive nanoparticles in cancer therapy. The pH of TME is lower than normal cells and therefore, it can be used for the development of pH‐sensitive nanocarriers. Furthermore, the presence of GSH can result in cleavage of bonds and cargo release; in addition to pH‐, enzyme‐, and GSH‐responsive nanocarriers, external stimuli‐responsive nanocarriers including light‐ and thermo‐sensitive nanoparticles can be developed to provide hyperthermia and to induce apoptosis in bladder cancer cells

In recent years, the application of smart and advanced nanoplatforms in cancer therapy has stirred much interest.^159^, ^160^, ^161^, ^162^ TME has several unique properties that can be employed for the development of stimuli‐responsive nanocarriers. The responsiveness to pH, redox, and light are the most common types of stimuli utilized for designing novel and smart nanoparticles. The pH‐sensitive nanoparticles function based on the alterations in pH levels of normal and cancer cells. The pH in normal cells and physiological media is larger than 7, while cancer cells demonstrate a pH lower than 6 that can be decreased based on the proliferation rate of tumor cells, glycolysis, and production of waste materials. Therefore, various kinds of bonds that are sensitive to pH such as hydrazone, acetal, and cis‐acotinyl are employed for the synthesis and preparation of pH‐sensitive nanocarriers. The pH‐sensitive nanoparticles are extensively applied for targeted delivery of cargo (drug or gene) at tumor site to promote accumulation of anti‐cancer agents in cancer cells. Redox is another stimulus for the synthesis of smart nanocarriers, and exposure to GSH or ROS can lead to cleavage of disulfide bond and release of cargo at tumor site. In addition to redox and pH, normal and cancer cells have differences in terms of levels of oxygen. Healthy cells are normoxic and have high levels of oxygen (70 mmHg), while tumor cells are hypoxic with low levels of oxygen (5 mmHg). Therefore, it is possible to use hypoxia as a stimulus for release of cargo at tumor site. Another approach (less common but still effective) is designing smart nanocarriers sensitive to enzymes. For instance, there are matrix metalloproteinases (MMPs) such as MMP‐2, MMP‐3, MMP‐9, MMP‐11 and MMP‐12 as enzymes in TME can cleave linkages to release cargo from nanocarriers. These are the endogenous factors that can be employed for the preparation of stimuli‐responsive nanocarriers. There are also efforts in synthesizing smart nanoparticles that are sensitive to external stimuli such as light. Exposure of such nanocarriers to light leads to release of cargo due to increase in temperature. It is of importance that normally, cancer cells have higher temperature compared to other cells that can be utilized for the synthesis of thermo‐sensitive nanocarriers.^163^, ^164^ The current section focuses on the application of smart nanocarriers for bladder cancer therapy (Figure 8)

Curcumin and Zn^2+^ have been processed into colloidal dispersions to prepare nanocomplexes with a high biocompatibility that would overtly increase the therapeutic index of cargo via enhancing cellular uptake. Furthermore, this nanocomplex promotes bioavailability of curcumin as a hydrophobic drug and it can be used for co‐delivery of curcumin and siRNA. The nanocomplexes were spherical in shape and had particle size of 80–500 nm with rough surface that is desirable for attachment of siRNA. Exposure of nanocomplexes to TME led to the release of curcumin in response to acidic pH and these positively charged nanocarriers effectively delivered siRNA to bladder tumor cells while protecting it against enzymatic degradation. Besides, these nanocarriers mediated endosomal escape of ElF5A2‐siRNA to release it in cytoplasm for downregulation of ElF5A2 as a tumor‐promoting factor to suppress proliferation and invasion of bladder tumor cells and to stimulate apoptosis via Bax upregulation and Bcl‐2 downregulation.^165^ The pH‐sensitive liposomes were employed for the purpose of bladder cancer therapy. The mPEG5kDa‐DSPE and stearoyl‐PEG‐polySDM that have pKa of 7.2, were used for synthesis of liposomes and then, bovine serum albumin (BSA) as a protein model was loaded into nanoparticles. The resulting nanocarriers demonstrated particle size of 287, 235, and 167, and the interaction of liposomes with MB49 cells and macrophages was higher in pH 6.5 compared to pH 7.4. The liposomes released protein in response of acidic pH, and they effectively delivered cargo to bladder epithelium, demonstrating their role as promising nanocarriers in cancer therapy.^166^ Most of the experiments have focused on pH‐sensitive nanocarriers in bladder cancer therapy. Multifunctional nanoparticles containing IONPs have been prepared with a core for providing magnetic characteristic, DOX as an anti‐cancer drug and calcium phosphate (CaP) as shell to mediate pH‐sensitive feature. Besides, nanomaterials were functionalized with RGD‐alginate to enhance their selectivity toward bladder tumor cells. These nanocarriers demonstrated high biocompatibility with particle size of 20 nm, and released the cargo (DOX) in response to change in pH and significantly enhanced internalization of chemotherapeutic agent in bladder tumor cells to provide both PTT and chemotherapy in synergistic cancer therapy.^118^

**FIGURE 9:**
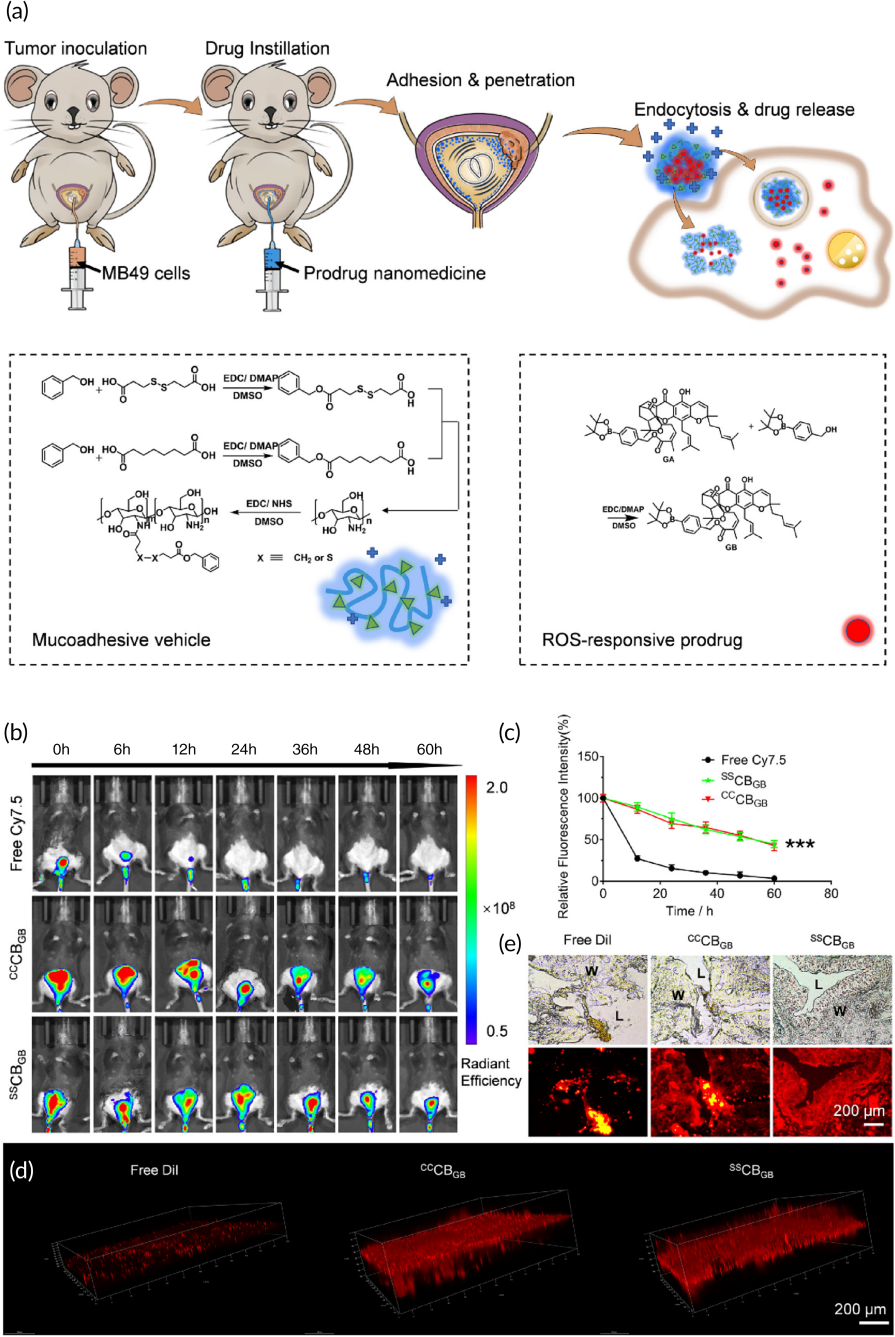
(a) Preparation of GSH‐responsive nanocarriers and their internalization in bladder tumor cells. (b) In vivo imaging. (c) Time‐related fluorescent intensity in bladder region. (d) 3D fluorescent distribution and (e) The fluorescence distribution of DiI or DiI‐labeled nanoparticles in the cross‐section of bladder urothelium. Reprinted with permission from Xu et al.^168^ from Elsevier

Furthermore, redox‐responsive nanocarriers were developed for bladder cancer therapy. The polypeptide nanogels were prepared from PLL‐P(LP‐co‐LC) polymers with disulfide links. 10‐Hydroxycamptothecin (HCPT) was loaded into core of nanogels via a facile diffusion resulting in high drug loading efficiency. These nanogels increased residence time and promoted penetration of cargo into bladder cancer tissues. The presence of GSH cleaved disulfide links to increase release of cargo and to enhance anti‐proliferative activity against bladder cancer.^167^ Another experiment developed positively charged chitosan‐polymeric nanoparticles for delivery of gambogic acid as a prodrug. Exposure to ROS and GSH resulted in the release of drug to selectively suppress progression of bladder tumor cells (Figure 9).^168^ The chitosan‐polymeric nanoparticles were internalized into bladder cancer cells via endocytosis and by providing stimulus‐responsive behavior, they prudentially accumulated at bladder tumor sites (Figure 9).^168^ Therefore, disulfide bonds lead to the formation of redox‐responsive nanocarriers in bladder cancer therapy^169^ and suppress progression.^170^

Taken together, these studies demonstrate that both pH‐ and redox‐responsive nanocarriers are promising smart nanoarchitectures for bladder cancer therapy. However, some limitations exist including relatively low examples examined, demanding more evaluation of efficacy in delivery for other anti‐cancer agents. Furthermore, there is no report on the application of external stimuli‐responsive nanocarriers which can be the focus of future studies. The strong potential of stimuli‐responsive nanocarriers in vivo (animal models) is their high accumulation at bladder cancer site.

## 8 NANOPLATFORMS IN BIOSENSING AND BIOIMAGING

The previous studies highlighted that nanoplatforms can provide effective treatment of bladder cancer and are potential agents for improving prognosis of patients. Noteworthy, nanoparticles can be employed for diagnosis of bladder cancer.^171^ The metal–organic frameworks (MOFs) are porous crystalline materials that have been composed mainly of metal ions/clusters and they have several great characteristics including high porosity, mechanical and thermal stability, the presence of organic groups, and large surface area.^172^, ^173^ MOFs have obtained their attention in the field of diagnosis in cancer. ZIF‐8‐based MOFs were designed for sensing telomerase activity in bladder cancer, where they were biomineralized with glucose oxidase (GOx)‐encapsulated ZIF‐8 (GZIF) and horseradish peroxidase (HRP), and this nanoplatform was capable of converting chemical energy to electricity to detect telomerase activity in bladder cancer tissues.^174^ The electrochemical sensing is an affordable technique and can be performed using portable devices with molecularly imprinted polymers (MIP) biosensing.^175^, ^176^, ^177^ The nuclear matrix protein 22 (NMP22) has been employed as a biomarker for diagnosis of bladder. Zinc oxide (ZnO) nanostructures with particle size of 214 nm were grown on sensing electrodes via a hydrothermal manner. This nanoplatform was used for electrochemical sensing of NMP22 in urine samples and in clinical trials, can detect this biomarker at a concentration range of 128–588 ng/ml.^178^

Another kind of nanoplatforms employed for biosensing is magnetic nanoparticles; however, their efficiency has been limited due to the adsorption of proteins on their surface which significantly reduces their specificity in biosensing application. The magnetic nanoparticles were prepared via precipitation reaction and then, their modification with PEI was performed to provide amine groups on the surface of nanostructures, leading to attachment of CBMA on magnetic nanostructures. The resulting nanoparticles had carboxylates on their surface that are of importance for purpose of biosensing. Compared to conventional nanoparticles such as PLGA or PEI nanostructures, CBMA‐PEI magnetic nanoparticles absorb lower level of proteins on their surface. Nuclear mitotic apparatus protein 1 (NuMA1) was employed as a biomarker, as it demonstrates overexpression in urothelial cancers and the nanoplatforms were able to detect this biomarker on the surface of bladder cancer cells. Therefore, more progress for its application in clinical trials should be performed.^179^

In addition to detection of biomarkers in bladder cancer, nanoplatforms can be employed for imaging of this malignant tumor. Although MRI, computed tomography, and positron emission tomography have been employed for diagnosis of bladder cancer, there is an absolute need for a diagnostic tool with high specificity, resolution, and deep tissue penetration. For this purpose, chitosan‐modified nanoparticles containing ferrimagnetic iron oxide nanocubes of 22 nm in size were developed. The reason for modification with chitosan was to improve the MRI contrast, and nanoarchitectures demonstrated high stability. These nanocarriers preferentially accumulated in bladder cancer tissue with poor biodistribution in other organs for tumor bioimaging.^180^ Notably, nanostructures can be employed in detection of biological markers for bladder cancer. From unprocessed urine, integrin alpha‐3 (ITGA3) can be sensed by nanostructures. For this purpose, europium‐doped nanoparticles coated with lectin were designed and such assay based on ITGA3 can distinguish bladder cancer from BPH.^181^ Another kind of nanoplatform for bladder cancer imaging is the application of quantum dots (QDs).^182^ Overall, QDs are promising agents in cancer imaging and theranostic purposes owing to their small size and high biocompatibility.^183^ Due to overexpression of CD47 on the surface of bladder tumor cells, surface of QDs was modified with anti‐CD47 to provide bladder cancer imaging. The QDs accumulated in bladder tumor and showed high safety profile. Furthermore, intravesical administration of QDs prevented the side effects of systemic administration. However, more investigation about long‐term toxicity of these nanomaterials for their clinical application should be performed.^182^

## 9 CANCER CELL INTERNALIZATION

Although different kinds of treatment modalities have been employed for bladder cancer, their low accumulation at tumor site is a troublesome problem. For instance, both synthetic drugs and phytochemicals are extensively used in bladder cancer treatment, their internalization in tumor cells is low and limits their therapeutic index. Same phenomenon occurs for nucleic acid drugs and their efficacy in gene expression regulation is restricted. According to our previous discussions, nanoparticles significantly enhance cellular uptake of therapeutic modalities in bladder cancer cells to augment their anti‐cancer activity. The current section shows the pathway followed by nanocarriers for improving the internalization of therapeutic modalities in bladder cancer.

**FIGURE 10:**
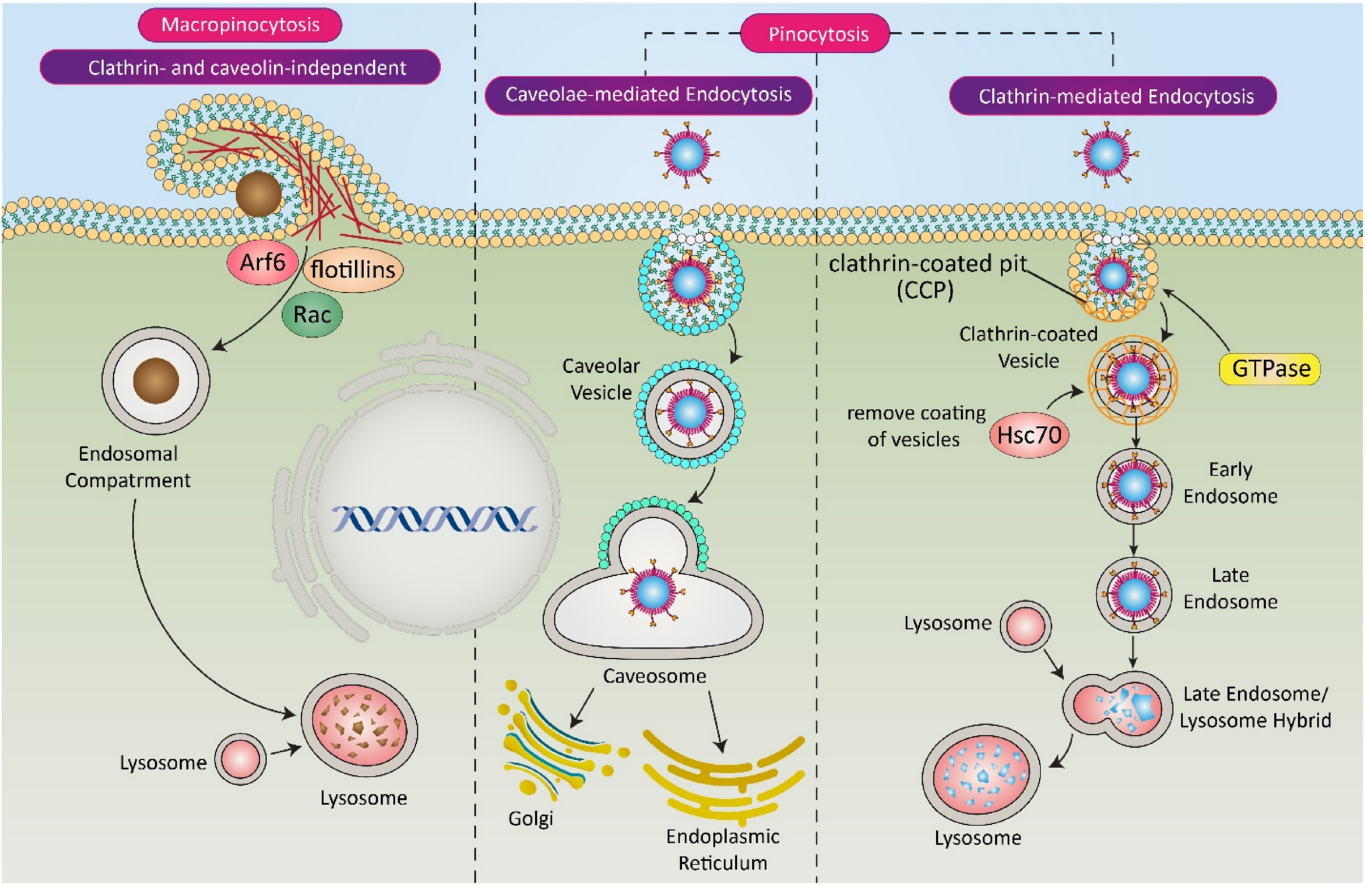
Various kinds of endocytosis pathways. Macropinocytosis and pinocytosis are two major pathways of endocytosis. Pinocytosis is divided into caveolae‐ and clathrin‐mediated endocytosis. In this mechanism, nanoparticles are integrated with cell membrane to internalize in cells, and then, they release their cargo

There are specific receptors on the surface of cells that are responsible for uptake of nanostructures through a mechanism, known as endocytosis.^184^ Nanomaterials have size and dimensions equivalent to intracellular organelles, and they may affect biological processes. Due to the interaction between living organisms and nanoparticles, cellular physiology is greatly affected and this interaction can be positive or negative.^185^ Both modified and nonmodified nanomaterials can be internalized by cells via endocytosis which starts with the generation of membrane invaginations and intracellular vesicles.^186^, ^187^ The pinocytosis and phagocytosis are two major kinds of endocytic pathway, and their difference is in the size of nanoparticles that they internalize. The particles with small size are internalized with pinocytosis, whereas large particles with sizes more than 500 nm are internalized via phagocytosis. Besides, pinocytosis is divided into clathrin‐mediated endocytosis, caveolae‐mediated endocytosis, and clathrin‐ and caveolae‐independent endocytosis.^188^ The clathrin‐mediated endocytosis and caveolae‐mediated endocytosis pathways are energy‐dependent and are responsible cellular uptake of biomolecules. The macropinocytosis is independent of clathrin‐mediated and caveolae‐mediated endocytosis, and they are driven by actin filaments (Figure 10).^189^, ^190^

The shape, size, and surface charge of nanoparticles are among those factors affecting internalization into bladder cancer cells. The surface modification of nanomaterials affects their ability in crossing over cell membranes and accumulation into bladder tumor cells. Hybrid nanomaterials from chitosan and HA were reported for delivery of an enzyme/pro‐drug complex containing HRP and indole‐3‐acetic acid (IAA) to suppress bladder cancer progression. The HA‐chitosan nanoparticles had particle size of 158 nm and their size increased to 170 and 200 nm due to loading of IAA and HRP, respectively. The zeta potential of nanomaterials was in the range of +20.36 to +24.40 mV, showing their high stability. Their encapsulation efficiency was up to 90% and they demonstrated high initial release (72%). These nanoparticles reduced the viability of bladder cancer cells by up to 88%.^191^ Although the internalization mechanism of hybrid nanoparticles in bladder tumor cells was not evaluated, due to modification of HA, they can selectively bind to CD44 receptors on the cell surface to internalize via receptor‐mediated endocytosis. The interaction between HA and CD44 has been confirmed in previous experiments.^192^, ^193^, ^194^ For instance, an experiment has prepared intravesical mucoadhesive hydrogels containing IONPs for reversing drug resistance in bladder cancer. The reason for using hydrogels is that IONPs cannot sufficiently accumulate at tumor site and in addition, increasing iron levels in IONPs promotes their efficacy in chemosensitivity via inducing ferroptosis. The IONPs were modified with HA and they were selectively internalized in bladder tumor cells overexpressing CD44 receptor via endocytosis.^195^ As a proof for the statement that both functional and nonfunctional nanomaterials can use endocytosis for internalization in bladder tumor cells, a study reported methotrexate‐loaded magnetic nanostructures are probably internalized via receptor‐mediated endocytosis.^196^ Although studies have shown the potential of nanoparticles for internalizing into bladder tumor cells via endocytosis,^197^ there are major gaps and limitations that should be addressed in future studies. Both modified and nonmodified nanocarriers can internalize in bladder cancer cells via endocytosis, however, only receptor‐mediated endocytosis has been investigated by studies and there should be experiments on clathrin‐ and caveolae‐mediated endocytosis for internalization of nanoparticles in bladder tumor cells. Furthermore, the evaluation of endocytosis is just on mechanism, but there is no report on the impact of size, shape, and surface charge of nanoparticles for internalizing in bladder cancer cells which should also be considered. Finally, the studies are limited to in vitro and when nanomaterials are introduced to blood circulation, a protein corona is formed on them that can affect their fate and internalization in bladder cancer cells, therefore in vivo tests need to be performed to further evaluate the internalization process of the nanoparticles by bladder cancer cells.

## 10 CLINICAL TRANSLATION

**FIGURE 11:**
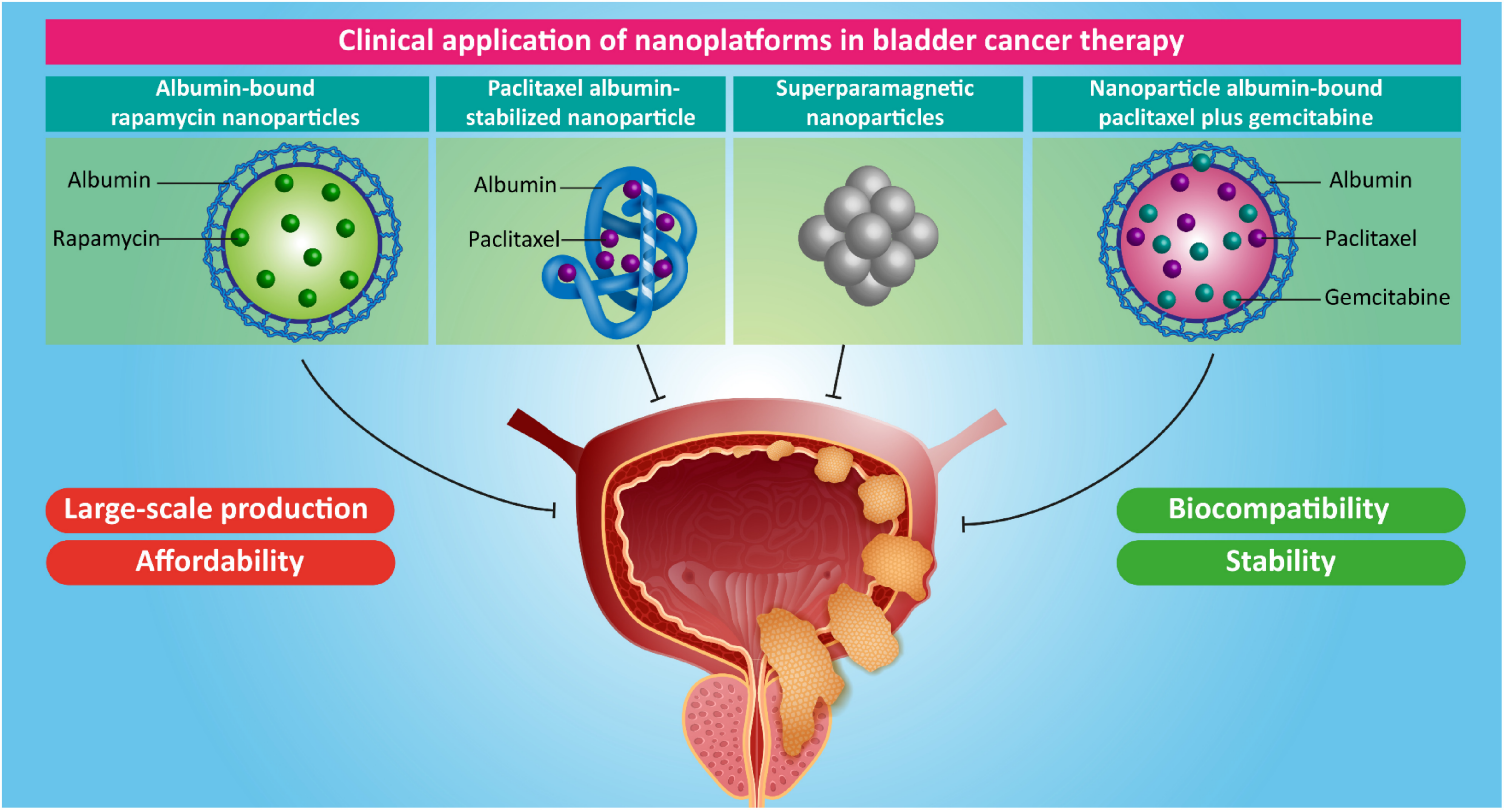
Clinical application of nanoplatforms in bladder cancer therapy with a focus on cons and pros. Nanostructures have been mainly recommended for purpose of drug delivery in bladder cancer treatment. There are some factors including large‐scale production, affordability, biocompatibility, and stability that should be considered for clinical application

Table 3 provides a summary of applications of nanostructures in clinics for treatment of bladder cancer patients. Nanomaterials have opened their way into clinics, however, there are still some issues that should be addressed. The number of clinical trials is low and no absolute conclusion about efficacy of nanomaterials or their long‐term biocompatibility in bladder cancer patients can be provided. Hence, probabilities and conclusions should be made based on preclinical studies to provide prospects for clinical trials that are going to be performed in near future. The first issue is related to safety profile of nanomaterials. Based on the experiments, various kinds of nanomaterials including polymeric‐, lipid‐, carbon‐, and metal‐based nanoparticles have been employed for the purpose of bladder cancer treatment and diagnosis. However, polymers can be degraded into monomers and the future studies should show how these monomers can affect biological events in cells and is there any serious adverse impact related to them? Furthermore, metal nanoparticles such as gold and IONPs can induce cell death in normal cells and their optimal concentration should be determined for future clinical applications. Interestingly, studies have shown that modification of nanomaterials with natural products such as chitosan and HA can improve biocompatibility of nanomaterials, which is promising. Studies have confirmed high stability of nanomaterials; therefore, they are of importance for improving the therapeutic index of chemotherapeutic agents by increasing their blood circulation time. The large‐scale production of nanomaterials and their affordability are other options that should be considered for their application in clinical trials for treatment of bladder cancer patients (Figure 11).

**TABLE 3:** The clinical trials using nanoparticles for the purpose of bladder cancer therapy

Treatment agent	Phase	Status	Reference
Nanoparticle albumin‐bound paclitaxel plus gemcitabine	Phase II	Terminated	NCT02887248
Paclitaxel albumin‐stabilized nanoparticle formulation	Phase II	Withdrawn	NCT02718742
Albumin‐bound rapamycin nanoparticles	Phase I	Completed	NCT02009332
Superparamagnetic nanoparticles	Not applicable (intervention study)	Terminated	NCT00147238

## 11 CONCLUSION AND REMARKS

Over the recent years, much attention has been geared toward treatment of urological cancers. Treatment of bladder cancer remains rather challenging for physicians, as tumor cells demonstrate abnormal proliferation and invasion, and in most cases in advanced stages, they have capacity of drug resistance development, resulting in chemotherapy failure. Therefore, novel methods should be used for treatment of bladder cancer which was summarized by this current review with a focus on nanomaterials. Overall, two kinds of drugs including phytochemical and synthetic drugs with anti‐cancer activity have been employed in treatment of bladder cancer. However, poor bioavailability of these compounds has restricted their clinical application. Although chemical modification of drug structure or drug repurposing have been considered as promising options in increasing bioavailability and preventing chemoresistance, respectively, these methods appear to not be enough for the complete eradiation of bladder cancer cells. Therefore, nanomaterials can significantly enhance internalization of anti‐cancer drugs in bladder tumor cells to potentiate their therapeutic index and reduce survival of cancer cells. Another application of nanomaterials is in gene delivery. Although siRNA, shRNA, and CRISPR/Cas are genetic tools that can be employed for bladder cancer therapy, their clinical application is almost impossible due to their drawbacks such as their degradation and accumulation at tumor site. Noteworthy, nanoparticles can provide a platform for co‐delivery of genes and drugs in synergistic therapy of bladder cancer.

The interactions in TME are responsible for the progression of bladder cancer. The macrophages are important and key players in cancer progression, but their regulation by nanomaterials in affecting bladder tumor malignancy has been ignored. Furthermore, studies related to nanomaterial‐mediated immunotherapy induction are limited that should be considered in future experiments about challenges and promises. Interestingly, nanoparticles can be employed for PDT and PTT, and in PDT, they enhance levels of ROS in TME to induce cell death. In PTT, an increase occurs in temperature (hyperthermia) to stimulate cell death. These two strategies not only reduce viability of bladder cancer cells, but also increase sensitivity of tumor cells to chemotherapy and immunotherapy. One of the progresses in treatment of bladder cancer is the development of smart nanocarriers. Redox‐ and pH‐sensitive nanoparticles can be designed for the purpose of bladder cancer therapy by release of cargo (gene or drug) at tumor site. Finally, it was reported that nanoparticles enter into bladder tumor cells via endocytosis.

Notably, nanostructures have been introduced to clinic in the treatment of bladder cancer. However, a few of them have been terminated and therefore, no absolute conclusion can be reached with regards to their efficacy in clinical. However, based on preclinical studies, the introduction of nanomaterials to treatment of bladder cancer is a promising strategy, and safety profile, affordability and large‐scale production of nanomaterials should be considered for broader clinical application.

## AUTHOR CONTRIBUTIONS

**Milad Ashrafizadeh:** Writing – original draft (lead); writing – review and editing (lead). **Jun Ren:** Writing – review and editing (supporting). **Yavuz Nuri Ertas:** Writing – review and editing (supporting).

## FUNDING INFORMATION

This work was supported by the Natural Science Foundation of Hubei Province (2019CFB591 to Zhaowu Ma), and the Bureau of Science and Technology of Jingzhou Municipality (2020CB21‐35 to Zhaowu Ma). Alan Prem Kumar was supported by a grant from the Singapore Ministry of Education (MOE‐T2EP30120‐0016).

## CONFLICT OF INTEREST

The authors declare no conflict of interest.

### PEER REVIEW

The peer review history for this article is available at https://publons.com/publon/10.1002/btm2.10353.

## Data Availability

Data sharing is not applicable to this article as no new data were created or analyzed in this study.
